# Lysosomal Ion Channels and Transporters: Recent Findings, Therapeutic Potential, and Technical Approaches

**DOI:** 10.1089/bioe.2025.0010

**Published:** 2025-03-18

**Authors:** Artem Kondratskyi, Andre Bazzone, Markus Rapedius, Rocco Zerlotti, Bastien Masson, Nidish Ponath Sadanandan, Joanne L. Parker, Alexandre Santinho, Marine Moutia, Abdou Rachid Thiam, Arlene Kemp, Fitzwilliam Seibertz, Nicoletta Murciano, Søren Friis, Nadine Becker, Alison Obergrussberger, Maria Barthmes, Cecilia George, Michael George, David Dalrymple, Bruno Gasnier, Simon Newstead, Christian Grimm, Niels Fertig

**Affiliations:** ^1^ Nanion Technologies GmbH, Munich, Germany.; ^2^ Oria Bioscience, Paris, France.; ^3^ Walther Straub Institute of Pharmacology and Toxicology, Faculty of Medicine, Ludwig Maximilians University, Munich, Germany.; ^4^ Department of Biochemistry, University of Oxford, Oxford, UK.; ^5^ The Kavli Institute for Nanoscience Discovery, University of Oxford, Oxford, UK.; ^6^ Laboratoire de Physique de l’École Normale Supérieure, ENS, Université PSL, CNRS, Sorbonne Université, Université Paris Cité, Paris, France.; ^7^ SB Drug Discovery a Sygnature Discovery Business, West of Scotland Science Park, Glasgow, UK.; ^8^ Saints-Pères Paris Institute for the Neurosciences, Université Paris Cité, Centre National de la Recherche Scientifique, Paris, France.; ^9^ Immunology, Infection and Pandemic Research IIP, Fraunhofer Institute for Translational Medicine and Pharmacology ITMP, Munich/Frankfurt, Germany.

**Keywords:** ion channels, transporters, pumps, lysosome, electrophysiology, patch clamp, APC, SSME

## Abstract

In recent years, there has been a growing interest in lysosomal ion channels and transporters due to their critical role in maintaining lysosomal function and their involvement in a variety of diseases, particularly lysosomal storage diseases, cancer, and neurodegenerative disorders. Recent advancements in research techniques, including manual and automated patch clamp (APC) electrophysiology, solid-supported membrane-based electrophysiology (SSME), and fluorescence-based ion imaging, have further enhanced our ability to investigate lysosomal ion channels and transporters in both physiological and pathological conditions, spurring drug discovery efforts. Several pharmaceutical companies are now developing therapies aimed at modulating these channels and transporters to improve lysosomal function in disease. Small molecules targeting channels like transient receptor potential mucolipin (TRPML) 1 and TMEM175, as well as drugs modulating lysosomal pH, are currently in preclinical and clinical development. This review provides an overview of the role of lysosomal ion channels and transporters in health and disease, highlights the cutting-edge techniques used to study them, and discusses the therapeutic potential of targeting these channels and transporters in the treatment of various diseases. Furthermore, in addition to summarizing recent discoveries, we contribute novel functional data on cystinosin, TRPML1, and two-pore channel 2 (TPC2), utilizing both SSME and APC approaches.

## Introduction

Since their discovery by Christian de Duve in the 1950s, lysosomes have emerged as central organelles responsible for the degradation and recycling of cellular waste. Initially identified as digestive compartments, lysosomes contain a highly acidic lumen (pH ∼4.6) that houses over 50 hydrolytic enzymes. These enzymes break down a wide array of macromolecules, including proteins, lipids, nucleic acids, and polysaccharides, into simpler molecules that can be reused by the cell. Beyond their degradative functions, lysosomes play a broader role in cellular homeostasis, including nutrient sensing, membrane repair, calcium signaling, and the regulation of autophagy. As such, lysosomes are now recognized not only as the cell’s recycling center but also as dynamic hubs of cellular metabolism and signaling.^1–3^

A key aspect of lysosomal function is the regulation of its internal environment, which is tightly controlled by various ion channels, transporters, and pumps present on the lysosomal membrane. These membrane transport proteins regulate the movement of ions, sugars, amino acids, and other solutes, which is critical for maintaining the acidic conditions required for enzymatic activity, as well as for ensuring proper lysosomal membrane potential.^4^ But the role of lysosomal channels, transporters, and pumps extends beyond lysosomal function, influencing cell-wide signaling pathways, particularly those involved in calcium homeostasis and nutrient sensing through key regulators like the mechanistic target of rapamycin (mTOR) and transcription factor EB (TFEB).^5^

Recent studies have revealed that dysregulation of lysosomal ion or solute transport can lead to profound cellular dysfunctions. Ion imbalance in lysosomes has been implicated in a wide range of diseases, including lysosomal storage disorders (LSDs), cancer, and neurodegenerative conditions such as Alzheimer’s and Parkinson’s diseases.^1^ In LSDs, mutations in proteins related to lysosomal ion homeostasis disrupt normal degradation processes, leading to the accumulation of undigested substrates within the lysosome, which results in cellular toxicity and, ultimately, organ failure.^6^ In neurodegenerative diseases, impaired lysosomal degradation of misfolded proteins and damaged organelles contributes to the progressive degeneration of neurons.

Despite the growing recognition of the importance of lysosomal channels and transporters, their biophysical properties and specific roles within the cell have only recently begun to be elucidated.^5,7^ With advancements in experimental methodologies, researchers have been able to explore the functions of transport proteins in greater detail. The development of lysosomal patch clamp has enabled direct recordings from native enlarged lysosomes, providing valuable insights into ion channel function in their physiological membrane environment.^8–10^ Recent breakthroughs in high-throughput automated patch clamp (APC) systems using purified lysosomes now allow for electrophysiological studies at a scale previously deemed unattainable.^11^ Solid-supported membrane (SSM)-based electrophysiology has further broadened our understanding of lysosomal ion channels and transporters by enabling precise current measurements in untreated lysosomes.^12,13^ Additionally, fluorescence-based ion imaging techniques, which employ ion-specific probes and genetically encoded indicators, provide dynamic and spatial insights into lysosomal pH, ion concentrations, and fluxes in live cells.^14,15^ Complementing these functional studies, structural approaches such as cryo-electron microscopy (cryo-EM) have unveiled the molecular architecture of lysosomal transport proteins, offering key insights into their mechanisms of action.^16,17^ Together, these tools are driving innovative research and therapeutic development targeting lysosomal transport proteins.

Given the vital role of lysosomal ion channels and transporters in maintaining cellular health and the increasing focus on their involvement in disease, this review aims to provide an update on the recent findings related to the lysosomal ion channels, transporters, and pumps in both physiological and pathological contexts. We will also discuss the cutting-edge techniques used to study lysosomal transport proteins and explore the emerging therapeutic potential of targeting these pathways to treat LSDs, cancer, and neurodegenerative diseases.

## Lysosomal Ion Channels, Pumps, and Transporters

Lysosomal ion channels, pumps, and transporters work synergistically to regulate the lysosome’s internal environment, enabling its diverse functions in degradation, signaling, and homeostasis.^4^ In recent years, the identification and characterization of several key ion channels and transporters have expanded our understanding of their roles in health and disease. The following sections highlight key lysosomal membrane transport proteins, discussing recent findings and providing examples of their functions and involvement in disease.

### Ion channels

Lysosomal ion channels are vital in maintaining lysosomal homeostasis and function. They are responsible for regulating the organelle’s ionic composition, pH, and membrane potential, which are essential for proper lysosomal function and cellular signaling.^5,6^

Recent advances in electrophysiological techniques and molecular biology have significantly expanded our understanding of lysosomal ion channels. These channels exhibit diverse properties in terms of ion selectivity, gating mechanisms, and regulatory factors. Their dysfunction has been implicated in various pathological conditions, ranging from LSDs to neurodegenerative diseases.^18^

In this section, we will explore the major ion channels found in lysosomes, including the transient receptor potential mucolipin (TRPML) channels and the two-pore channel 2 (TPC2), which are crucial for calcium signaling; the chloride channel CLN7, which plays a role in lysosomal acidification; and the potassium channel TMEM175, which contributes to the regulation of lysosomal membrane potential and pH stability. Additionally, we will review channels primarily recognized for their roles at the plasma membrane, but which also localize to lysosomal membranes and play important roles in autophagy, lysosomal calcium signaling, and membrane trafficking. These include P2X4, BK, volume-regulated anion channels (VRAC), TMEM63A, TRPM2, TRPA1, CACNA1A, cystic fibrosis transmembrane conductance regulator (CFTR), and TWIK2. We will highlight recent discoveries and ongoing research that continue to reshape our understanding of lysosomal ion channels and their potential as therapeutic targets.

#### TRPML

The TRPML family consists of three members in mammals: TRPML1, TRPML2, and TRPML3. These channels are primarily localized to endolysosomal membranes and play crucial roles in various cellular processes, including lysosomal Ca^2+^ signaling, membrane trafficking, and ion homeostasis. While sharing some common features, each TRPML channel has unique expression patterns, regulatory mechanisms, and physiological functions.^19^

TRPML1, also known as MCOLN1, is the most extensively studied member of the family. It is ubiquitously expressed in all mammalian cell types and is primarily localized in late endosomes and lysosomes (LELs).^20^ TRPML1 is a nonselective cation channel permeable to Ca^2+^, Na^+^, K^+^, Fe^2+^, and Zn^2+^.^10,21^ It is activated by phosphatidylinositol 3,5-bisphosphate [PI(3,5)P2], a LEL-resident phosphoinositide, and inhibited by plasma membrane PI(4,5)P2, suggesting compartment-specific regulation.^22,23^ TRPML1 plays critical roles in various lysosomal functions, including Ca^2+^ release, membrane fusion and fission, autophagosome-lysosome fusion, lysosome trafficking, and exocytosis.^5^ Numerous studies have reported the involvement of TRPML1 in autophagy, describing its role in modulating TFEB and mTOR pathways.^24^

Mutations in the *TRPML1* gene cause mucolipidosis type IV (MLIV), a neurodegenerative lysosomal storage disorder.^25^ Beyond MLIV, TRPML1 is also implicated in Parkinson’s disease, Alzheimer’s disease, other LSDs and cancer. In cancer, TRPML1 enhances tumor invasiveness by regulating lysosomal exocytosis, particularly in pancreatic ductal adenocarcinoma.^26^ Recent findings also identified TRPML1 as a key player in ferroptosis resistance in cancer cells, offering a novel target for enhancing the efficacy of cancer therapies.^27^

The development of TRPML1 modulators continues to be a major research area, with ongoing efforts to discover more specific and potent activators and inhibitors. Several pharmaceutical companies have TRPML1 modulators in their pipelines, including Merck, Lysoway Therapeutics, Casma Therapeutics, Threebrooks Therapeutics, Nine Square Therapeutics, and Tenvie Therapeutics.^28–33^ Small-molecule activators (ML-SA1, MK6-83) and inhibitors (ML-SI, estradiol analogs EMDE, PRU-10, PRU-12) have been shown to affect autophagy, providing tools for developing a possible treatment for neurodegenerative diseases.^34^ In addition, gene therapy approaches targeting MCOLN1 have shown promise in preclinical models of MLIV, correcting neurological deficits and improving motor function.^35^

TRPML2 (or MCOLN2) is primarily found in immune cells, where it is localized in both LELs and recycling endosomes and is involved in regulating ion homeostasis, membrane trafficking, and innate immune responses. Like TRPML1, TRPML2 is activated by PI(3,5)P2 and is involved in Ca^2+^ signaling. However, TRPML2 has unique features, such as its sensitivity to osmotic changes. Hypotonic challenge and physical pressure on endolysosomal membranes can potentiate agonist-evoked TRPML2 currents, suggesting a role in osmoregulation of membrane trafficking.^36^ In some cell types, TRPML2 may have redundant functions with TRPML1, particularly in lysosomal regulation.^19^

TRPML2 is associated with various diseases, particularly infectious diseases and cancers. TRPML2-mediated magnesium deprivation has been shown to restrict the replication of *Salmonella Typhi*, illustrating its role in host defense through nutritional immunity.^37^ Furthermore, TRPML2 expression is involved in modulating viral infections. For example, agonists of TRPML2, such as ML2-SA1, were shown to impair the replication of Zika and hepatitis E viruses by disrupting intracellular cholesterol distribution and genome replication.^38^

In glioblastoma, TRPML2 expression correlates with poor prognosis and resistance to the chemotherapy drug temozolomide. High TRPML2 expression in glioblastoma stem cells has been linked to reduced survival rates, and targeting TRPML2 in these cells has emerged as a potential therapeutic strategy.^39^

TRPML3 (mucolipin 3, MCOLN3) is another member of the TRPML family, which is found in both LELs and early endosomes. TRPML3 is not ubiquitously expressed like its relative TRPML1 and is primarily described in alveolar macrophages, melanocytes, cochlear hair cells, and uroepithelial cells. Recent studies have also identified TRPML3 in various glands, including the parathyroid, thyroid, salivary, adrenal, and pituitary glands, as well as in the testes and ovaries, indicating that the channel may play important roles in hormone secretion or uptake.^40^

Like TRPML1, TRPML3 has been shown to regulate lysosomal calcium, lysosomal trafficking, and autophagy.^41^ However, in contrast to TRPML1, which is activated by acidic luminal pH, TRPML3 is inhibited under low pH conditions, a property that allows it to remain inactive in typical lysosomal environments but become activated in response to lysosomal alkalization, such as during pathogen invasion, thereby facilitating processes like exosome release and bacterial clearance.^42^

Gain-of-function mutations in TRPML3 have been associated with deafness and pigmentation defects in mice, while its dysfunction has been implicated in emphysema and chronic obstructive pulmonary disease.^43,44^

Several small molecules have been developed to specifically modulate TRPML3 activity. Among the activators, SN-2, EVP-21, and ML3-SA1 stand out for their selective activation of TRPML3. While effective activators exist, no specific inhibitors for TRPML3 have been identified, with current options affecting other TRPML isoforms as well.^34^

#### TMEM175

In recent years, transmembrane protein 175 (TMEM175) has gained attention as a promising therapeutic target for Parkinson’s disease.^45,46^ This lysosomal ion channel is critical for maintaining lysosomal pH and function. Initially characterized as a potassium leak channel, recent findings suggest that TMEM175 primarily functions as a proton-activated, proton-selective channel under physiological lysosomal conditions.^47^

TMEM175 activity is tightly regulated to ensure proper lysosomal function. Luminal protons act as endogenous activators, increasing H^+^ currents and positioning TMEM175 as a proton-activated lysosomal channel that maintains pH homeostasis through a negative-feedback loop.^47^ Arachidonic acid can also activate TMEM175 H^+^ currents, potentially serving as a regulatory signal for lysosomal pH. Furthermore, protein interactions with lysosomal-associated membrane protein 1 (LAMP-1), LAMP-2, protein kinase B (AKT), and B-cell lymphoma 2 (Bcl-2) have been reported to regulate TMEM175 function, with AKT activating the channel and LAMP-1, LAMP-2, and Bcl-2 exerting inhibitory effects.^48–50^

Structural analyses have shown that human TMEM175 is a dimeric channel with 12 transmembrane domains, and TMEM175 currents have been studied in detail through various techniques, including automated whole-cell patch clamp, lysosomal patch clamp, as well as solid-supported membrane-based electrophysiology (SSME).^13,51^

TMEM175 is ubiquitously expressed in mammalian cells and is particularly important in neurons, where it facilitates the efficient lysosomal degradation of aggregated proteins.^47^ TMEM175 dysfunction leads to impaired lysosomal degradation pathways, resulting in the accumulation of toxic protein aggregates like α-synuclein and increased neuronal vulnerability.

Genetic evidence from genome-wide association studies highlights TMEM175′s relevance to common sporadic forms of Parkinson’s disease, making it a highly promising and broadly applicable therapeutic target.^45,52^

Beyond Parkinson’s disease, TMEM175 has been implicated in bone disorders, with reduced expression or activity impairing osteoblast differentiation and leading to decreased bone mineralization and increased fracture risk.^53^ Emerging research highlights the broader implications of TMEM175 in immune responses and infectious diseases through its regulation of endolysosomal pathways. It has been shown that TMEM175 mediates the antitoxin activity of broad-spectrum antipathogenic compounds like dimethyl-ABMA (DABMA) by regulating endolysosomal trafficking, highlighting its role in host–pathogen interactions and positioning it as a potential druggable target for anti-infective therapies.^54^ Moreover, TMEM175 has been linked to the tumor immune microenvironment, particularly in cancers like oral squamous cell carcinoma, where it correlates with immune deregulation and poor outcomes.^55^

Given TMEM175's significant role in disease, particularly Parkinson’s disease, there is growing interest in developing selective modulators of its activity as potential therapeutic agents. Synthetic agonists such as (4-(2-Butyl-6,7-dichloro-2-cyclopentyl-indan-1-on-5-yl) oxobutyric acid (DCPIB) and ML 67-33 have been identified as activators of TMEM175, while 4-aminopyridine (4-AP) has been shown to act as a low-affinity inhibitor.^47,56^ Additionally, recently discovered selective inhibitors such as 2-phenylpyridin-4-ylamine (2-PPA) and AP-6, may serve as valuable tools to study the roles of TMEM175 in regulating lysosomal function and provide useful templates for future therapeutic development in Parkinson’s disease.^57^

Interestingly, most of the pharmaceutical companies that have TRPML1 modulators in their pipelines are also developing TMEM175 modulators, emphasizing the growing recognition of lysosomal ion channels as promising therapeutic targets and highlighting the strategic focus on exploiting lysosomal function to address unmet clinical needs.

#### TPC2

Two-pore channels (TPCs) are a ubiquitously expressed family of endolysosomal cation channels comprising two members in humans: TPC1 and TPC2.^58^ Unlike TPC2, which is mainly localized to LELs, TPC1 is primarily found in early endosomes and will therefore not be a topic of this review.^59^

TPC2 is a phosphoinositide-activated Na^+^-selective channel that demonstrates significant Ca^2+^ permeability when activated by agonists like nicotinic acid adenine dinucleotide phosphate (NAADP) through auxiliary NAADP-binding proteins, JPT2 and Lsm12.^60^ Recent cryo-EM studies revealed conformational changes in TPC2’s selectivity filter, underlying its plasticity in ion conductance and gating mechanisms.^61^ Unlike TPC1, TPC2 operates independently of pH variations and lacks apparent voltage sensitivity.^62^

Along with JPT2 and Lsm12, TPC2 activity is fine-tuned by multiple other regulatory inputs. Key regulators include the adenosine triphosphate (ATP)/mammalian target of the rapamycin (mTOR) pathway, which suppresses TPC2-mediated Na^+^ flux; auxiliary proteins like Rab7a, which enhance TPC2 activation; and TMEM63A/B, which antagonizes TPC2 activity.^63–65^

TPC2 is critical for lysosomal functions and supports processes like autophagy and lysosomal exocytosis.^66^ Ca^2+^ release through TPC2 can trigger global calcium signaling through functional coupling between TPC2 and inositol trisphosphate receptors (IP3Rs), enabling coordinated lysosomal-ER calcium signaling and amplifying its impact on cellular processes.^67^

Dysregulated TPC2 activity contributes to a variety of diseases, from neurodegenerative and infectious diseases to cancer and cardiovascular disorders.^68^ As such, TPC2 represents a promising therapeutic target for a range of diseases, with inhibitors showing potential in treating viral infections, neurodegenerative disorders, and possibly certain cancers. For instance, TPC2 inhibition has been reported to reverse pronounced lysosomal morphology defects in fibroblasts from Parkinson’s disease patients with the common LRRK2 G2019S mutation.^69^ In cancer, increased TPC2 activity, often driven by regulators like Rab7a, promotes tumor growth, invasion, and metastasis, particularly in melanoma models.^64^ Loss of TPC2 leads to cholesterol buildup and fatty liver disease, while gain-of-function mutations cause pigmentation defects.^70–76^ TPC2 is also critical for Ebola and coronavirus entry and escape from lysosomes, making it a potential target for antiviral therapies.^77,78^

Pharmacological modulation of TPC2 holds significant promise for treating diseases linked to lysosomal dysfunction. Thus, a number of synthetic modulators have been developed for TPC2. Compounds such as TPC2-A1-N and TPC2-A1-P provide tools to selectively modulate TPC2’s ion selectivity and function. While TPC2-A1-N enhances Ca^2+^ release for processes like lysosomal exocytosis, TPC2-A1-P shifts the channel toward Na^+^ selectivity (while still remaining permeable to Ca^2+^), potentially addressing lysosomal osmotic balance. Additionally, inhibitors like verapamil, Ned19, tetrandrine, naringenin, pratensein (MT-8), and duartin (UM-9) along with tetrandrine derivatives such as SG-005 and SG-094, block TPC2-mediated Na^+^ currents, offering therapeutic strategies for conditions involving excessive TPC2 activity. However, translating these findings into clinical applications requires careful consideration of potential off-target effects, given TPC2’s involvement in diverse physiological processes.^66^

#### CLN7

Initially considered to be a putative transporter, CLN7 (also known as MFSD8) has recently been identified as an endolysosomal chloride channel that plays a crucial role in regulating lysosomal function.^79,80^ This protein, encoded by the *MFSD8* gene, is a 518-amino acid polytopic lysosomal transmembrane protein with 12 membrane-spanning domains and therefore is a unique chloride channel with no sequence similarity to other known chloride channels.

CLN7 mediates outward-rectifying Cl^−^ currents that are sensitive to chloride channel blockers, diisothiocyanatostilbene disulfonic (DIDS), NFA, and NPPB. It conducts other halide anions, with higher permeability to iodide, and does not rely on other ions or ATP for Cl^−^ transport. The channel’s activity is regulated by pH, with decreased luminal pH significantly increasing CLN7 currents.^80^

CLN7 has been shown to regulate lysosomal membrane potential, lysosomal pH, as well as lysosomal calcium content. It was suggested that CLN7 promotes lysosomal calcium release through a TRPML1-dependent mechanism, thereby affecting various intracellular signaling pathways. Overexpression of CLN7 induces endolysosomal enlargement in a Ca^2+^/calmodulin-dependent way.^80^

Mutations in CLN7 cause a variant form of late-infantile neuronal ceroid lipofuscinoses (vLINCL, also known as Batten disease), characterized by severe neurological symptoms, including visual deficits, motor problems, and frequent seizures.^81^ Studies on animal models, including mice, dogs, and rabbits with *MFSD8* mutations, demonstrate neurodegenerative phenotypes and provide insights into disease mechanisms.^82–84^ Knocking out CLN7 leads to pathological features similar to those observed in vLINCL patients, including retinal degeneration, accumulation of autofluorescent lipofuscin, lysosomal dysfunction, impaired autophagy, and neurodegeneration.

Recent progress in therapeutic approaches has provided new opportunities for addressing CLN7-associated disorders. One notable development is the use of personalized antisense oligonucleotide (ASO) therapy, exemplified by Milasen, which was designed specifically for a patient with a unique MFSD8 mutation.^85^ Also, gene replacement strategies using viral vectors to deliver functional CLN7 have demonstrated efficacy in preclinical models, showing improved lysosomal function and reduced neurodegeneration.^86^ These advances clearly highlight the potential of tailored genetic therapies in addressing CLN7-related pathologies.

### Other lysosomal ion channels

Certain ion channels that are primarily recognized for their roles at the plasma membrane have been found to also localize to lysosomal membranes in some cell types. These dual-localized ion channels play versatile roles in cellular physiology, with their functional properties and regulatory mechanisms often influenced by their subcellular context. At the lysosomal membrane, these channels contribute to ion homeostasis, hydrolase activation, membrane potential, exocytosis, membrane fusion, and trafficking, whereas at the plasma membrane, they participate in processes such as signaling, volume regulation, or ion transport. These channels include P2X4, BK, VRAC, TMEM63A, TRPM2, TRPA1, CACNA1A, CFTR, and TWIK2.

#### P2X4

P2X receptors are ligand-gated, nonselective cation channels that open in response to ATP binding. In humans, there are seven P2X receptor subtypes (P2X1–P2X7), which can form either homomeric or heteromeric trimers. Among these, P2X4 is unique in that, in addition to being expressed on the plasma membrane, it is also localized to endolysosomal membranes.^87^

At the plasma membrane, P2X4 receptors are rapidly and constitutively internalized and trafficked to endolysosomes, from which they can be recycled back to the cell surface. The balance between dynamic trafficking to and from the plasma membrane or retention within endolysosomes depends on cellular context.^88^

It has been proposed that targeting P2X4 receptors to acidic organelles is essential for their re-sensitization. Cycling through acidic compartments enables the protonation and deprotonation of key histidine residues in the extracellular loop of P2X4, a mechanism critical for restoring receptor sensitivity to ATP.^89^

However, several studies suggest that P2X4 receptors also play a physiological role within the lysosomal compartment. First, it has been demonstrated that P2X4 forms functional ATP-activated cation channels on lysosomal membranes and that it is regulated by luminal pH. Patch-clamp studies on enlarged lysosomal vacuoles revealed that P2X4 activity was inhibited by acidic luminal pH but was activated when the luminal pH increased in the presence of ATP.^90^ The same group subsequently demonstrated that calcium release through lysosomal P2X4 activates calmodulin (CaM) and promotes endolysosomal membrane fusion, an effect that was prevented by the inhibition of either P2X4 or CaM.^91^

A few studies indicated that lysosomes contain high levels of ATP (up to millimolar range), transported into lysosomes by the vesicular nucleotide transporter (VNUT)/SLC17A9 using V-ATPase-generated voltage gradient as the driving force. However, acidic luminal conditions prevent premature activation of lysosomal P2X4 by ATP, suggesting a functional interaction between V-ATPase, SLC17A9, and P2X4 in lysosomal membranes.^92^ Therefore, an alkalinizing shift in lysosomal pH should be sufficient to activate lysosomal P2X4 receptors and influence Ca^2+^/CaM-dependent lysosomal membrane trafficking.

Some recent studies highlighted the role of lysosomal P2X4 receptors in disease. In murine models of experimental autoimmune encephalomyelitis (EAE), P2X4 activation in microglia promotes an anti-inflammatory phenotype, enhancing myelin recovery through efficient endolysosome fusion.^93^ Ivermectin, a P2X4 potentiator, potentiated myelin phagocytosis, promoted the remyelination response, and ameliorated clinical signs of EAE. Similarly, P2X4 facilitates lysosomal exocytosis in hepatocytes after partial hepatectomy, aiding liver regeneration by increasing ATP in bile and preventing bile-induced damage.^94^

#### BK

BK channels, also known as Maxi-K, SLO1, Kca1.1, or KNMCA1, are ubiquitously expressed large-conductance K^+^ channels that could be found both at the plasma membrane and intracellular organelles, including mitochondria, ER, nucleus, or lysosomes.^95–97^ These channels are activated by membrane depolarization and elevated cytosolic Ca^2+^ concentrations and exhibit large conductance of 100–300 pS. This dual activation allows BK channels to integrate changes in intracellular calcium and membrane potential, making them important negative-feedback systems in various physiological processes.^98^

Lysosomal BK (Lyso-BK) channels have been detected in various cell types, including fibroblasts, neurons, myoblasts, and astrocytes. Their lysosomal localization is determined by two dileucine sorting motifs in the channel’s cytoplasmic carboxy terminus.^96^

The functional importance of lysosomal BK channels has been explored in various systems. It has been shown that these channels play a key role in regulating lysosomal calcium homeostasis, membrane potential, and trafficking.

Lyso-BK channels regulate both the release and uptake of lysosomal Ca^2+^. They are functionally coupled with TRPML1 and TRPML3 channels and facilitate TRPML1/TRPML3-mediated Ca^2+^ release and membrane trafficking.^41,96^ For example, functional coupling between BK and TRPML1 has been shown to regulate large particle phagocytosis through modulating lysosomal exocytosis in macrophages.^99^ Downregulation of BK channels leads to increased lysosome volume and accumulation of membranous inclusions, resembling LSDs. Conversely, BK upregulation can mitigate cellular phenotypes of several LSDs.^100^ Likewise, BK overexpression rescued the impaired TRPML1-mediated Ca^2+^ release and abnormal lysosomal storage in cells from Niemann-Pick C1 patients.^96^ In line with these results, activation of TRPML3/BK/mTOR positive feedback loop has been shown to promote autophagy and bacterial clearance.^41^

Interestingly, along with facilitating Ca^2+^ release from lysosomes, Lyso-BK channels are also required for efficient refilling of lysosomal Ca^2+^ stores, supposedly via ER–lysosome membrane contact sites.^95^

#### LRRC8

LRRC8 proteins, initially identified as components of VRACs on the plasma membrane, have recently been discovered to also play a crucial role in lysosomal function.^Leucine-rich repeat-containing protein 8 (LRRC8), initially identified as a component of volume-regulated anion channels (VRACs) on the plasma membrane, has recently been discovered to also play a crucial role in lysosomal function.101,102^ Members of the LRRC8 family are expressed on lysosomal membranes in various mammalian cell types, where they form functional lysosomal VRACs (Lyso-VRACs) and generate significant anion currents in response to low cytoplasmic ionic strength conditions.^101^

Lyso-VRAC currents are essential for the formation of large lysosome-derived vacuoles, which store excess water and later release it through exocytosis. This process ensures the maintenance of cytosolic water balance and enables lysosomes in mammalian cells to function as the cell’s “bladder,” protecting against necrotic cell death caused by physiological stresses such as hypoosmotic, hypoxic, and hypothermic conditions.^101^

Beyond their critical role in lysosomal osmoregulation, a recent preprint has highlighted that Lyso-VRACs also regulate mTOR signaling, lysosomal morphology, pH, and autophagic flux in primary skeletal myotubes.^103^

Overall, further research is required to elucidate the precise mechanisms by which the lysosomal LRRC8 regulates lysosomal function. Recent evidence that VRACs can mediate the transport of second messengers, metabolites, antibiotics, and anticancer drugs suggests that Lyso-VRACs in lysosomes may have roles that extend well beyond osmoregulation.^104^

#### TMEM63

TMEM63 proteins are a family of mechanosensitive ion channels found in animals, with three members (TMEM63A, B, and C) present in mammals. These channels function as high-threshold mechanosensors, characterized by small conductance.^105^

Recently, several studies reported the intracellular expression of TMEM63 at the membranes of different organelles, including endolysosomes, lamellar bodies, and secretory granules.^63,106,107^

One study suggested that Drosophila TMEM63 as well as mouse TMEM63A are localized at the lysosomal membrane where they function as intrinsic mechanosensors of lysosomes and modulate lysosomal morphology and function.^107^ TMEM63 mutant flies display impaired lysosomal degradation, synaptic loss, progressive motor deficits, and premature death, with several of these phenotypes mirroring symptoms observed in TMEM63-associated human diseases, including hypomyelinating leukodystrophy and hereditary spastic paraplegia. Notably, mouse TMEM63A has been shown to mediate lysosomal mechanosensitivity in Neuro-2a cells, highlighting its functional conservation in mammals.^107^

TMEM63A/B were also reported to be localized at the limiting membrane of the lamellar body, a lysosome-related organelle that stores pulmonary surfactant and ATP in alveolar type 2 epithelial cells.^106^ Stretch-induced activation of TMEM63A/B channels promoted the release of surfactant and ATP from lamellar bodies following fusion with the plasma membrane. The released ATP then initiated Ca^2+^ signaling in alveolar epithelial cells, which amplified lamellar body exocytosis. In contrast, the loss of TMEM63A/B leads to atelectasis and respiratory failure in mice due to impaired surfactant secretion.^106^

In pancreatic acinar cells, TMEM63A (also called OCaR1) has been shown to localize in the membrane of lysosomes and secretory granules, where it operates as a negative regulator of TPC1/2-mediated Ca^2+^ release from these organelles.^63^

#### TRPM2

Transient receptor potential melastatin 2 (TRPM2) is yet another plasmalemmal ion channel that has been reported to localize on lysosomal membranes in various cell types. Studies have shown that TRPM2 functions as a Ca^2+^-release channel on lysosomal membranes, activated by factors such as ADP-ribose and oxidative stress.^108–110^

In pancreatic β cells, TRPM2 was found to colocalize with lysosomal markers like LAMP-1, mediate Ca^2+^ release and contribute to oxidative stress-induced cell death.^110^ In vascular smooth muscle cells, TRPM2 partially colocalizes with lysosomes and facilitates autophagic degradation through lysosomal acidification. TRPM2 knockout substantially reduced both lysosomal acidification and autophagic degradation.^109^ In dendritic cells and macrophages, however, TRPM2 has been shown to predominantly localize on lysosomal membranes, where it regulates processes like dendritic cell maturation and chemotaxis.^108^

A recent study revealed a complex interplay between TRPM2, lysosomal Ca^2+^ release, endoplasmic reticulum (ER) Ca^2+^ refilling, and store operated calcium entry (SOCE) in the context of inflammasome activation and metabolic inflammation, highlighting TRPM2 as a key player in these processes.^111^ In bone marrow-derived macrophages, TRPM2 has been shown to function as a lysosomal Ca^2+^ efflux channel activated by mitochondrial reactive oxygen species (ROS) in response to lipopolysaccharide and palmitic acid stimulation. The TRPM2-mediated lysosomal Ca^2+^ release leads to delayed JNK activation, ASC phosphorylation, and subsequent inflammasome activation. TRPM2 knockout mice showed reduced inflammasome activation and metabolic inflammation in adipose tissue when fed a high-fat diet.^111^

#### TRPA1

Transient receptor potential ankyrin 1 (TRPA1), primarily known for its role as a nociceptor on the plasma membrane of dorsal root ganglion (DRG) neurons, has also been identified on lysosomal membranes. In DRG neurons, TRPA1 has been shown to colocalize with the lysosomal marker LAMP-1, particularly in regions near the plasma membrane. TRPA1 agonists, such as allyl isothiocyanate, effectively induced TRPA1-mediated Ca^2+^ release from acidic stores, generating lysosomal Ca^2+^ sparks. Notably, the study shows that lysosomal TRPA1 contributed significantly to Ca^2+^-dependent vesicle exocytosis, neuropeptide/calcitonin gene-related peptide (CGRP) release from DRG neurons, and pain sensation in mice.^112^

However, it should be noted that another group was unable to replicate the findings of this study. They argued that the observed lysosomal TRPA1-mediated calcium transients and CGRP release from DRG neurons are more likely attributable to the influx of insufficiently buffered extracellular calcium rather than release from lysosomal stores.^113,114^

#### CACNA1A

While voltage-gated Ca^2+^ channels (VGCCs) are mostly known to localize to the plasma membrane, recent evidence suggests that some VGCCs, particularly the P/Q-type (CACNA1A, Cav2.1), play a crucial role in lysosomal function and neuronal homeostasis.^115,116^

Two studies have shown that VGCCs are localized on lysosomal membranes in both mice cerebellar neurons (CACNA1A) and photoreceptor neurons from fruit flies (cac), where they regulate the fusion of autophagic vacuoles with lysosomes through their calcium channel activity.^115,116^ The α1 subunit CACNA1A and the α2δ subunit (cac and straightjacket in Drosophila) of VGCCs are essential for this process. Loss of these subunits leads to autophagic defects in both Drosophila and mice.

Importantly, the lysosomal CACNA1A, but not the plasma membrane-resident CACNA1A, is required for lysosomal fusion with endosomes and autophagosomes, and the function of lysosomal CACNA1A in autophagy is distinct from its plasma membrane role in synaptic transmission. CACNA1A mutant neurons exhibit reduced lysosomal calcium storage, ultimately resulting in axonal degeneration in the cerebellum. Consequently, the authors suggested that dysfunction of lysosomal VGCCs may contribute to certain neurodegenerative conditions.^115,116^

#### CFTR

The CFTR is primarily recognized as a plasma membrane chloride channel critical for maintaining epithelial ion and fluid homeostasis. However, emerging evidence reveals that in multiple cell types, CFTR is also localized to intracellular organelles, including lysosomes.^117–120^

In macrophages, CFTR plays a crucial role in regulating lysosomal acidification, especially in the context of autophagy and bacterial clearance. It has been shown that CFTR is recruited to LC3-labeled autophago-lysosomes harboring bacteria such as *Burkholderia cenocepacia*, contributing to their acidification and improving autophagic flux and bacterial clearance. CFTR modulators, such as tezacaftor/ivacaftor, improved autophagy flux, lysosomal acidification, and bacterial clearance in cystic fibrosis macrophages, suggesting potential therapeutic utility.^117^

In line with this study, CFTR has been also shown to participate in phagosomal pH control and bacterial killing capacity. Alveolar macrophages from *CFTR*^−/−^ mice exhibited defective killing of internalized bacteria, despite retaining the ability to phagocytose and generate an oxidative burst. The absence of CFTR led to impaired acidification of lysosomes, resulting in an environment conducive to bacterial replication.^120^

CFTR’s function in lysosomal acidification appears to be more significant in reacidifying alkalinized lysosomes rather than maintaining baseline pH levels. This finding has important implications for diseases associated with lysosomal alkalinization, such as Stargardt’s disease, again suggesting that CFTR modulation could be a potential therapeutic approach.^119^

It’s important to note, however, that the role of CFTR in lysosomal acidification has been a subject of particular interest and debate in the field of cystic fibrosis research, with different labs reporting conflicting results. Some studies have failed to show any effect of CFTR on lysosome acidification, potentially suggesting that this effect could be context-dependent or cell-type specific.^121,122^

#### TWIK2

TWIK2, a member of the two-pore domain potassium (K2P) channel family, is primarily known for its weak background potassium currents at the plasma membrane. However, recent studies have revealed that at rest TWIK2 is predominantly expressed in LAMP-1-positive lysosomal compartments and endosomes.^123^

The channel contains specific sequence signals that target it to lysosomes: a tyrosine-based motif and two di-leucine-like motifs. Sequential inactivation of these trafficking motifs progressively abolishes TWIK2’s targeting to lysosomes and promotes its relocation to the plasma membrane.

Electrophysiological studies demonstrate that TWIK2 functions as a potassium channel in lysosomal membranes, producing outwardly rectifying K^+^ currents that likely contribute to maintaining lysosomal membrane potential and pH homeostasis. This potassium conductance supports lysosomal acidification by counterbalancing the H^+^-pumping activity of the V-ATPase.

Interestingly, TWIK2 expression has been shown to affect lysosomal number and size, with cells expressing TWIK2 displaying increased lysosomal biogenesis. This suggests that TWIK2 plays an active role in regulating lysosomal dynamics.^123^

In the following table, we present a concise overview of key lysosomal ion channels, highlighting their primary functions and disease associations (Table 1). To the best of our knowledge, this table includes most, if not all, currently known lysosomal ion channels. However, with advancements in techniques, the number of identified lysosomal ion channels may continue to grow. One potential candidate is the SIDT2 protein, which was previously reported to function as a lysosomal cation-conducting protein.^124^ However, some researchers suggest that SIDT2 primarily acts as a transporter for nucleic acids, particularly dsRNA and DNA, while others propose that it may function as a dsRNA receptor in the endocytic pathway rather than as a direct transporter or channel.^125^ Further research is needed to clarify its precise role.

**Table 1.** Lysosomal Ion Channels

Target	Function	Associated diseases/pathologies	References
TRPML1 (MCOLN1)	Calcium release, lysosomal trafficking, autophagy	Mucolipidosis IV, Parkinson’s, Alzheimer’s, cancer	^5,10,21–27^
TRPML2 (MCOLN2)	Calcium signaling, immune response regulation	Infectious diseases, cancer, immune disorders	^19,36–39^
TRPML3 (MCOLN3)	Lysosomal calcium release, membrane trafficking	Hearing loss, pigmentation defects, COPD	^40–44^
TMEM175	Lysosomal pH homeostasis, protein degradation	Parkinson’s, bone disorders, immune dysfunction	^45–57^
TPC2	Sodium and calcium transport, lysosomal signaling	Parkinson’s, cancer, viral infections, pigmentation defects	^58–78^
CLN7 (MFSD8)	Chloride channel, lysosomal acidification, calcium release	Batten disease, neurodegeneration	^79–86^
P2X4	ATP-gated cation channel, calcium signaling, membrane fusion	Autoimmune disorders, liver regeneration	^87–94^
BK	Large-conductance K+ channel, lysosomal calcium regulation	Lysosomal storage disorders, neurodegeneration	^41,95–100^
VRAC (LRRC8)	Volume-regulated anion channel, lysosomal osmoregulation	Lysosomal dysfunction, osmoregulation disorders	^101–104^
TMEM63A/B	Mechanosensitive ion channel, lysosomal function	Neurodegeneration, lysosomal function impairment, hypomyelinating leukodystrophy, hereditary spastic paraplegia	^63,105–107^
TRPM2	Lysosomal calcium release, oxidative stress response	Metabolic inflammation, oxidative stress disorders	^108–111^
TRPA1	Nociception, lysosomal calcium release, exocytosis	Neuropathic pain, inflammatory conditions	^112–114^
CACNA1A (Cav2.1)	Voltage-gated Ca2+ channel, autophagy regulation	Neurodegeneration, lysosomal dysfunction	^115,116^
CFTR	Chloride transport, lysosomal acidification, immune response	Cystic fibrosis, lysosomal storage diseases	^117–122^
TWIK2 (K2P)	Potassium channel, lysosomal pH balance	Lysosomal storage disorders, acidification defects	^123^

CFTR, cystic fibrosis transmembrane conductance regulator; COPD, chronic obstructive pulmonary disease; TPC, two-pore channel; TRPML, transient receptor potential mucolipin; VRAC, volume-regulated anion channels.

### Pumps

Pumps are primary active transporters that consume energy from a primary source to move substrates across membranes. In humans, the most diverse and relevant pumps are ATPases, which use energy from ATP hydrolysis for active transport.

ATPases can be categorized into several classes, including P-, F-, and V-type ATPases, with the V-ATPase being the key proton pump of the lysosome. Additionally, ATP-binding cassette (ABC) transporters, which also rely on ATP hydrolysis, facilitate the transport of a diverse range of substrates, including lipids and drugs, often at a slower rate.^126,127^

Lysosomal pumps play a critical role in maintaining the ionic environment necessary for proper lysosomal function. They also participate in cellular signaling and ion homeostasis, impacting various physiological processes and disease states. The dysfunction of lysosomal pumps has been implicated in a range of disorders, from neurodegenerative diseases to metabolic syndromes.

In the following sections, we will explore key lysosomal pumps—including V-ATPase, ATP13A2, and ATP7B—their roles in health and disease, and their therapeutic potential.

#### The vacuolar H^+^-ATPase

Perhaps the most well-known membrane pump in lysosomes is the vacuolar H^+^-ATPase (V-ATPase), a large, multisubunit proton pump that actively transports H^+^ ions into the lysosomal lumen.^128^

The V-ATPase consists of two main domains: the cytoplasmic V1 domain, responsible for ATP hydrolysis, and the membrane-bound V0 domain, which facilitates proton translocation across the membrane. The V1 domain comprises multiple protein subunits (A–H), while the V0 domain contains subunits that form a rotary motor essential for driving proton transport into the lysosomal lumen.^128^

This canonical function of V-ATPase is essential for the proper degradation of macromolecules delivered to lysosomes either endocytically or via autophagy.^129^ However, in addition to its primary function, V-ATPase also has noncanonical roles in cellular signaling, including coordinating responses to nutrient availability and energy stress,^130,131^ or regulating Wnt and Notch pathways.^129,132^

Lysosomal V-ATPase dysfunction has been implicated in various disorders, particularly neurodegenerative (Alzheimer’s disease, Parkinson’s disease, amyotrophic lateral sclerosis) and LSDs.^133^ Mutations in V-ATPase subunits have been linked to progressive myoclonus epilepsy, developmental and epileptic encephalopathy, autosomal recessive cutis laxa type II, and other pathologies.^134–136^

Furthermore, plasma membrane V-ATPases have been shown to promote tumor cell survival and invasiveness, while dysregulation of plasma membrane V-ATPases in several cell types has been linked to infantile osteopetrosis, recessive distal renal tubular acidosis, and sensorineural deafness.^137^

In addition, very recent findings associated ATP6V1D, a V-ATPase subunit, with cancer stem cell self-renewal and progression via lysosome acidification-dependent and independent mechanisms, suggesting novel therapeutic avenues in hepatocellular carcinoma.^138^ In obesity, V-ATPase subunits, such as v1a, are critical for adipocyte differentiation, with knockdown studies demonstrating their involvement in cholesterol and steroid metabolism.^139^ Also, V-ATPase inhibitors are now being explored as eco-friendly alternatives to pesticides in agricultural settings.^140^

Nevertheless, despite the ongoing efforts in developing selective inhibitors/activators and understanding the complex roles of V-ATPases in different cellular processes, to our knowledge, currently, there are no V-ATPase-targeting drugs approved for clinical use on the market. A few compounds showed promise in preclinical studies (including inhibitors: bafilomycin A/D, concanamycin A, cleistanthin A, SB 242784, FR167356, diphyllin; activators: EN6; expression enhancers: NCH-51, rifampicin, DNLA) but did not advance to clinical trials.^129,133^

Developing drugs that specifically target V-ATPases in disease-relevant tissues or cells remains challenging. The multisubunit structure of V-ATPases makes it difficult to design highly specific inhibitors, whereas nonselective inhibition can lead to high systemic toxicity due to the ubiquitous presence of V-ATPases in cellular processes.

#### ATP13A2

Another ATPase in lysosomes is ATP13A2 (also known as PARK9), a P5-type ATPase involved in the transport of polyamines, supporting degradation pathways and protecting cells from environmental stress, including oxidative stress and heavy metal toxicity.^141^ ATP13A2 consists of 10 transmembrane helices, three conserved cytoplasmic domains (N, A, and P domains) that coordinate to hydrolyze ATP, and two specific extensions (C-terminal domain and N-terminal domain).^142^

Recent evidence suggests ATP13A2 acts not only as a polyamine transporter but also as a lysosomal H^+^,K^+^-ATPase, exporting K^+^ from the lysosomal lumen to the cytoplasm while importing H^+^ into lysosomes.^143^ Thus, like V-ATPase, ATP13A2 also contributes to maintaining acidic lysosomal pH.

Some earlier studies suggested ATP13A2 might also transport Mn^2+^ and Zn^2+^ ions, but more recent structural and functional analyses have not supported this idea. However, ATP13A2 does contribute to reducing the toxicity of these ions. The exact mechanism for this protective effect is not fully understood but is at least in part related to the antioxidative and metal-chelating effects of polyamines.^144^

The dysfunction in ATP13A2 disrupts lysosomal function, ion homeostasis (particularly for metals such as Mn^2+^, Fe^3+^, and Zn^2+^), and autophagic degradation of misfolded proteins like α-synuclein, leading to progressive neuronal degeneration.^145,146^

ATP13A2 is primarily associated with Kufor-Rakeb syndrome, a form of juvenile-onset Parkinson’s disease, but is also implicated in a range of other neurodegenerative disorders, such as hereditary spastic paraplegia, neuronal ceroid lipofuscinosis, multiple system atrophy, and amyotrophic lateral sclerosis.^147^

Beyond its neurological functions, ATP13A2 has been recently identified as a regulator of innate immune responses, particularly in plasmacytoid dendritic cells. ATP13A2 plays a central role in TLR9/7 activation in human pDCs by regulating endolysosomal pH and mitochondrial ROS generation.^148^

Recent therapeutic approaches focus on restoring ATP13A2 function or compensating for its loss. Compounds such as ginsenoside Rg1 have shown promise in alleviating ATP13A2-related lysosomal dysfunction and reducing neurodegeneration in Parkinson’s disease models under stress conditions.^149^ Furthermore, gene therapy approaches targeting ATP13A2 (lentivirus-mediated ATP13A2 silencing) have successfully induced Parkinson’s-like neurodegeneration in primate models, providing a platform for further exploration of therapeutic strategies.^150^ New experimental platforms like iSCORE-PD, which utilize isogenic stem cells carrying ATP13A2 mutations, have been developed to study its function and disease mechanisms in human-relevant models.^151^

#### ATP7B

Along with lysosomal-resident V-ATPase and ATP13A2, a copper-transporting P-type ATPase, ATP7B, normally residing in the trans-Golgi network, has been reported to redistribute to the lysosomal membrane in response to elevated copper levels in hepatocytes. It plays a key role in preventing copper toxicity by promoting lysosomal exocytosis to release excess copper into the bile.^152^

Recent findings, however, indicate that ATP7B can localize to lysosomes even in the absence of elevated copper levels. This suggests that ATP7B may have broader physiological roles in lysosomal function beyond copper export.^153^

In line with this, ATP7B has been shown to interact with autophagy machinery, particularly LC3 proteins, suggesting a potential cooperative role in copper clearance through autophagy.^154^

Dysfunction in ATP7B is central to the pathophysiology of Wilson disease, a disorder that leads to copper accumulation in the liver and brain. ATP7B-deficient cells are unable to properly excrete copper through lysosomes, resulting in toxic copper buildup. Therefore, enhancing ATP7B’s lysosomal function or improving its trafficking could help mitigate copper overload in Wilson disease.^152^

At present, along with approved copper chelators, new experimental models and small-molecule correctors of ATP7B trafficking are being explored as potential treatments to restore copper homeostasis in affected cells.^155,156^

Beyond Wilson’s disease, ATP7B has been demonstrated to be implicated in cancer cell resistance to platinum-based chemotherapies. Overexpression of ATP7B is linked to the vesicular sequestration of platinum compounds, reducing their cytotoxic effectiveness and identifying a novel approach to treating certain cancers.^157^

The table below gives a brief summary of the main lysosomal pumps, their key functions, and related diseases (Table 2). Please note that this table is not exhaustive and includes only the pumps discussed above. Lysosomes contain a broader array of pumps, including ABC transporters (e.g., ABCA2, ABCA3, ABCA5, ABCB6, ABCB9, ABCD4). For more information on lysosomal ABC transporters, see recent reviews on the topic.^158^

**Table 2.** Lysosomal Pumps

Target	Function	Associated diseases/pathologies	References
V-ATPase	Vacuolar H^+^-ATPase, lysosomal acidification, metabolic regulation	Neurodegeneration, lysosomal storage diseases, cancer	^128–140^
ATP13A2 (PARK9)	Polyamine-transporting P5-type ATPase, lysosomal acidification, oxidative stress protection	Parkinson’s, Kufor-Rakeb syndrome, neurodegeneration, immune regulation	^141–151^
ATP7B	Copper-transporting P-type ATPase, heavy metal detoxification	Wilson’s disease, heavy metal toxicity, cancer	^152–157^

### Uniporters, symporters, and antiporters

The electrochemical gradient generated by lysosomal ATPases drives the transport of ions as well as various small molecules, such as nucleotides and amino acids, through a set of specialized transporters, each tailored to transport specific substrates and maintain lysosomal function. These transporters can be classified into uniporters, symporters, and antiporters, based on how substrate translocation is coupled to the electrochemical gradient, and they are collectively known as porters.

In humans, porters belong to the solute carrier (SLC) superfamily, the second-largest membrane protein family after G protein-coupled receptors (GPCRs). It comprises over 450 members organized into 66 families, with diverse structures and functions.^159^ The structure of SLC transporters varies widely, with predictions suggesting anywhere from 3 to 14 transmembrane domains. This structural diversity reflects the broad range of substrates transported by SLC proteins, including amino acids, sugars, nucleotides, inorganic ions, and various metabolites.

In lysosomes, SLC transporters play essential roles in nutrient sensing, metabolic regulation, and the maintenance of cellular homeostasis. So far, over 30 lysosomal SLC transporters have been identified and studied for their roles in health and disease. It should be noted, however, that most of these transporters are also localized in other parts of cells, such as plasma membrane, endoplasmic reticulum, endosomes, mitochondria, secretory vesicles, and others, meaning they can have other nonlysosomal functions.^7^

Lysosomes harbor a vast repertoire of transporters, and the breadth of this topic makes it impractical to cover all of them in a single review. Instead, we have chosen to focus on a subset of transporters, selected based on their well-characterized roles, therapeutic relevance, or the novelty and uniqueness of their mechanisms. This focused approach enables a deeper exploration of specific mechanisms and provides insights into lysosomal biology while highlighting the diversity and importance of these transporters in health and disease.

For readers seeking a more comprehensive understanding of lysosomal transporters, several excellent reviews are available.^4,7,18,160,161^

#### SLC15A3 (PHT2) and SLC15A4 (PHT1)

SLC15A3 and SLC15A4 are proton-coupled amino acid transporters primarily localized in endolysosomal membranes of immune cells. Both play crucial roles in innate immunity and inflammatory responses by mediating the transmembrane transport of histidine, various di- and tripeptides, and certain bacterial peptidoglycans from lysosomes to the cytosol.^162^

SLC15A3 is transcriptionally regulated by key factors such as hypoxia-inducible factor 1-alpha (HIF1α) and nuclear factor kappa B (NF-κB), which are activated during inflammatory conditions. These factors promote SLC15A3 expression, exacerbating inflammatory processes, particularly in conditions like ischemic stroke, where it drives neuroinflammation by polarizing microglial cells toward a pro-inflammatory M1 phenotype.^163^ SLC15A3 is also upregulated through Toll-like receptor (TLR) signaling pathways, further linking it to immune system activation.^164^ Furthermore, SLC15A3 has been implicated in conditions like pulmonary fibrosis, where it modulates macrophage oxidative stress responses, highlighting its role in chronic inflammatory diseases.^165^ These studies suggest that targeting SLC15A3 could offer therapeutic benefits in inflammatory diseases, oxidative stress-related conditions, and cancer. Inhibition of SLC15A3 has shown promise in reducing inflammation and oxidative damage, especially in pulmonary fibrosis and ischemic stroke models, where blocking SLC15A3 can attenuate disease progression.^163,165^

Additionally, SLC15A3 has been associated with antiviral responses. It is induced in immune cells during viral infections, such as herpes simplex virus-1, where it enhances the production of type I and type III interferons via the mitochondrial antiviral signaling protein (MAVS) and stimulator of interferon genes (STING) pathways, helping to control viral replication.^166^

SLC15A4, like SLC15A3, is integral to immune cell function. It plays a pivotal role in immune signaling, especially through its interaction with TLRs and nucleotide-binding oligomerization domain-containing protein (NOD) signaling pathways, which are crucial for pathogen detection and immune activation.^167,168^

SLC15A4 is essential for optimal TLR signaling, particularly TLR7, TLR8, and TLR9-mediated type I interferon production in plasmacytoid dendritic cells. It acts as a signaling scaffold by recruiting the innate immune adapter TASL (TLR Adaptor interacting with SLC15A4 on the Lysosome) to endolysosomes, which is crucial for the activation of IRF5 and subsequent inflammatory responses.^168^

SLC15A4 is also required for mTOR-dependent IFN-I response to TLR stimulation. SLC15A4 loss disturbs endolysosomal pH regulation and likely affects V-ATPase integrity, leading to disruption of the mTOR pathway.^169^

SLC15A4 plays a critical role in systemic lupus erythematosus, colitis, and psoriasis, contributing to the production of pathogenic antibodies and regulation of IFN-I responses.^170,171^

The loss of SLC15A4 significantly ameliorates the symptoms of several diseases, and therefore SLC15A4 is increasingly recognized as a promising therapeutic target. Small-molecule inhibitors developed against SLC15A4, such as feeblin, effectively disrupt the SLC15A4-TASL module, leading to anti-inflammatory effects in preclinical models.^172^

A recent study identified a potent SLC15A4 inhibitor, AJ2–30, which effectively inhibited the activation of TLR7–9 signaling, inflammatory cytokine production, and SLC15A4-mediated NOD 1/2 activation both in primary human and mouse immune cells. Mechanistic studies revealed that AJ2–30 disrupts SLC15A4 interactions with components of the mTOR pathway, leading to impaired TLR-mediated signaling and subsequent cytokine production.^167^

#### SLC17A9 (VNUT)

Lysosomes hold a significant amount of ATP, which can be released via lysosomal exocytosis in response to various stimuli. The SLC17A9, also known as vesicular nucleotide transporter (VNUT), is an ATP transporter responsible for lysosomal ATP accumulation.^173^ SLC17A9 deficiency leads to reduced lysosomal ATP levels, compromised lysosome function, and ultimately results in cell death, supposedly due to the dysfunction of cathepsin D.^174^

Along with being localized on lysosomes, SLC17A9 also localizes to secretory vesicles and is essential for vesicular ATP release, playing a key role in purinergic signaling in various cell types, including neurons and immune cells. This makes SLC17A9 an important contributor to neurotransmission, inflammation, and immune responses, as well as nociceptive signaling.^175^ Astrocytic SLC17A9, for example, influences ATP release in the brain, modulating synaptic activity, anxiety, and depressive-like behavior.^176^ In bladder urothelium, SLC17A9-dependent ATP exocytosis plays a role in regulating bladder compliance and urine storage.^177^

SLC17A9 expression is often upregulated in various pathological conditions, such as cancer, inflammatory diseases, and neuropathic pain states. Recent research suggests it could be a potential therapeutic target for certain cancers. SLC17A9 has been implicated in the progression of hepatocellular carcinoma, colorectal cancer, non-small-cell lung cancer, and osteosarcoma. It promotes cancer stem cell-like properties and tumorigenesis in hepatocellular carcinoma and in cancers such as non-small-cell lung cancer; SLC17A9 correlates with poor prognosis and immune cell infiltration, indicating its role in tumor microenvironment modulation and potential as a biomarker.^178–181^

Besides cancers, mutations in SLC17A9 have been associated with disseminated superficial actinic porokeratosis,^182^ congenital heart defect (Tetralogy of Fallot),^183^ and susceptibility to phantom tooth pain.^184^

SLC17A9 inhibitors could potentially provide a novel therapeutic approach by reducing ATP release, thereby interrupting the signaling pathways involved in neuroinflammation or chronic pain without the side effects associated with opioids or nonsteroidal anti-inflammatory drugs.

Several small molecules targeting SLC17A9 have been used to study its physiological role. These include nonselective pan-SLC17 inhibitors, such as Evans blue, DIDS, and acetoacetate, as well as more selective eicosapentaenoic acid, atractyloside, and clodronate.^175^

Clodronate has been shown to reduce pain transmission and nociceptive signaling and could provide the pharmacological basis to develop new local analgesic drugs.^185^ Eicosapentaenoic acid, a potent SLC17A9 inhibitor, showed efficacy in treating neuropathic and inflammatory pain by inhibiting purinergic transmission.^186^

#### SLC17A5 (sialin)

SLC17A5, also known as sialin, is a lysosomal membrane transporter responsible for the export of free sialic acid from lysosomes into the cytoplasm after the lysosomal degradation of glycoproteins and glycolipids.^187^ It is unique among the solute carrier family 17 (SLC17) members for its dual transport mechanism: a proton-driven export of sialic acid or nitrate and a membrane potential-driven transport of certain neurotransmitters, such as aspartate, glutamate, and *N*-acetylaspartylglutamate (NAAG), into synaptic vesicles.^188–190^

Recent analyses have revealed the structural basis of this unique sialin’s dual transport mechanisms. The protein exhibits distinct conformational states, including apo cytosol-open, apo lumen-open, NAAG-bound, and inhibitor-bound structures.^191,192^ A positively charged cytosol-open vestibule accommodates either NAAG or the sialin inhibitor Fmoc-Leu-OH, while its luminal cavity potentially binds sialic acid.^192^

A recent study showed that in skeletal muscle, sialin acts as a nitrate transporter, contributing to the nitrate/nitrite reductive pathway for NO generation. This pathway becomes particularly important in conditions where the neuronal NO synthase (nNOS) enzyme is absent or dysfunctional.^193^

Mutations in the *SLC17A5* gene cause Salla disease and infantile sialic acid storage disorder, LSD characterized by the accumulation of sialic acid within lysosomes.^187,194^

Sialin dysfunction is also implicated in metabolic and neurological disorders, such as atherosclerosis and Parkinson’s disease, due to its role in modulating nitric oxide (NO) bioavailability and lysosomal function.^195,196^

Recently, small molecules like Fmoc-Leu-OH have been identified as inhibitors of sialin, providing tools to investigate its physiological roles and help develop pharmacological chaperones for Salla disease.^197^ Additionally, CRISPR-Cas9-based approaches, particularly adenine base editing, have been successfully used to correct specific pathogenic mutations in *SLC17A5*, offering potential therapeutic strategies for treating sialic acid storage disorders.^198^

#### SLC66A4 (cystinosin)

Cystinosin (encoded by the *CTNS* gene) is a proton-coupled cystine transporter, responsible for the export of cystine from lysosomes into the cytoplasm.^199^ The transporter has seven transmembrane helices and uses the proton gradient created by the V-ATPase to export cystine from the lysosome in a 1:1 ratio.^200–202^

Mutations in the *CTNS* gene can lead to cystinosis, a lysosomal storage disease characterized by an intralysosomal accumulation of cystine, the disulfide of the amino acid cysteine. Cystinosis primarily affects the kidneys, causing renal Fanconi syndrome and progressive renal failure, but also impacts other organs, leading to extrarenal complications like ocular damage, muscle wasting, endocrine disturbances, and neurological issues.^199,203^

Cystinosin’s role extends beyond lysosomes, as it has also been discovered in melanosomes and linked to the control and regulation of melanin.^204^ This finding correlates well with hypopigmentation reported in some cystinosis patients and suggests the potential implication of cystinosin in pigmentation disorders and melanoma.

Recent research has revealed an important connection between cystinosin and mTOR signaling, particularly in the context of kidney proximal tubule cells. Cystinosin has been shown to act as a “transceptor” at the lysosomal membrane, where it interacts with components of the V-ATPase and Ragulator-RRAG GTPase scaffold complex, signaling cystine sufficiency to mechanistic target of rapamycin complex 1 (mTORC1) and modulating its activation in response to nutrient availability, thus regulating cellular metabolism and differentiation.^202,205–207^

The current standard treatment for cystinosis is cysteamine, a cystine-depleting agent that can reduce cystine accumulation in lysosomes. However, cysteamine therapy does not fully reverse organ damage, particularly in the kidneys, and requires lifelong administration. Emerging research has explored new promising approaches, including gene therapy, mRNA-based therapies, and modulation of key molecular pathways like mTORC1 or NLRP2.^205,208–210^

#### ClC-7

ClC-7 is a member of the CLC chloride channel family, but it operates primarily as a 2Cl^−^/H^+^ antiporter rather than a classical chloride channel. It was proposed that this antiport activity supports the function of the V-ATPase and contributes to lysosomal acidification by forming the lysosomal counterion pathway.^211^

However, recent findings have shown that ClC-7 is largely inhibited by PI(3,5)P2 in normal cellular conditions, preventing it from strongly influencing baseline lysosomal pH. Instead, it is activated by stimuli that decrease PI(3,5)P2 concentrations, thereby providing additional Cl^−^ as a counterion and facilitating further lysosomal acidification.^212^

Structurally, ClC-7 forms homodimers, with each monomer containing an independent ion transport pathway. Moreover, it requires the beta-subunit OSTM1 for stability and correct localization within lysosomes.^213^

ClC-7 expression is ubiquitous, with particularly high levels in bone tissue and the central nervous system. In bone, ClC-7 is highly expressed in osteoclasts, where it is essential for bone resorption. Its function in neurons is also crucial, as it contributes to lysosomal function and cellular homeostasis in the nervous system.^214^

Interestingly, while loss-of-function and gain-of-function mutations in ClC-7 can cause diverse clinical phenotypes, such as osteopetrosis, neurodegenerative lysosomal storage disease, myelination defects, and albinism, their pathomechanisms share an impairment of lysosomal function and autophagic flux.^215,216^

Mutations in the *CLCN7* gene are linked to several forms of osteopetrosis, including autosomal recessive osteopetrosis, intermediate autosomal osteopetrosis, and autosomal dominant osteopetrosis type 2 (ADO2). These genetic mutations disrupt the function of ClC-7, leading to impaired bone resorption, increased bone density, and skeletal abnormalities.^217^ Furthermore, ClC-7 mutations have been implicated in neurodegenerative diseases due to their impact on lysosomal storage and neural cell function.^218^

Recent findings also revealed that downregulation of CLC-7 exacerbates pain in neuropathic conditions, providing a new target for developing therapies to treat chronic pain.^219^

Although all these studies highlight the therapeutic potential of targeting ClC-7 in metabolic bone diseases and neurodegeneration, currently, there are no widely used small molecules directly targeting ClC-7. SiSaf, a biopharmaceutical company known for its RNA therapeutics, is now developing SIS-101-ADO, a novel siRNA therapeutic that targets and suppresses the expression of the mutant *CLCN7* gene in ADO2 patients. This therapeutic has been recently granted the Orphan Drug Designation by the FDA.

#### TMEM165

Lysosomes have long been known as storage sites for calcium. However, while much is known about how Ca^2+^ is released from lysosomes, the mechanisms by which they refill their Ca^2+^ stores have remained unclear. Although pH-dependent calcium uptake as well as Ca^2+^/H^+^ exchange mechanisms were suggested, the specific proteins driving these processes remained unidentified.^220^

In a recent study, Zajac et al. identified the gene *lci-1* in *Caenorhabditis elegans* and its human homolog *TMEM165* as key players in the process of lysosomal calcium import.^221^

TMEM165 is predominantly localized in the Golgi apparatus, where it plays a crucial role in maintaining the ionic balance necessary for proper glycosylation processes. In the Golgi, TMEM165 is believed to act primarily as a Ca^2+^/H^+^ antiporter to regulate the Golgi’s lumenal environment. Emerging evidence suggests that TMEM165 plays a role in manganese (Mn^2+^) transport within the Golgi.^222,223^ Manganese is an essential cofactor for various Golgi-resident enzymes involved in glycosylation, and its dysregulation can impair glycosylation pathways.^224,225^

Unlike the previously assumed Ca^2+^/H^+^ exchanger role, recent findings positioned TMEM165 as a proton-activated Ca^2+^ importer in lysosomes (LCI), operating in a pH-dependent manner.^221^ It should be noted, however, that a very recent preprint by Chen et al. demonstrates that TMEM165 functions as Ca^2+^/H^+^ antiporter in lysosomes.^226^ Nevertheless, despite the difference in mechanism proposed by these groups, both studies suggest that TMEM165 is central to calcium import into lysosomes.

#### MFSD1

Major facilitator superfamily domain-containing protein 1 (MFSD1) is a ubiquitously expressed lysosomal membrane protein that functions as a general dipeptide uniporter in lysosomes. It forms a complex with glycosylated lysosomal membrane protein (GLMP) to facilitate the transport of cationic, neutral, and anionic dipeptides across the lysosomal membrane.^227^ Unlike SLC15 transporters, MFSD1 exhibits high selectivity for dipeptides. It fails to transport single amino acids and tripeptides.^227,228^

Knockout studies in mice have shown that loss of MFSD1 leads to significant physiological changes, including splenomegaly, liver dysfunction, and disrupted liver homeostasis.^229^ Recent research has also demonstrated that MFSD1, in complex with GLMP and GIMAP5 (GTPase of immunity-associated protein 5), plays an essential role in lymphocyte survival and supports lymphocyte development.^230^

In cancer, loss of MFSD1 correlates with enhanced tumor cell migration and metastasis due to alterations in β1 integrin recycling and activation. Conversely, increased MFSD1 expression levels are associated with improved prognosis in patients with lung, breast, and gastric tumors.^231^

Future research directions may include investigating the potential therapeutic applications of modulating MFSD1 function in liver pathologies, immune disorders, and cancer.

#### LYCHOS

LYCHOS (LYsosomal CHOlesterol Signaling, also known as GPR155) is a lysosomal transmembrane protein with a unique fusion architecture, combining a GPCR-like domain with a transporter-like domain.^12^ It functions as a cholesterol sensor that links cholesterol abundance to the activation of the mTORC1, a key regulator of cell growth and metabolism.^232^ The transporter-like domain of LYCHOS shares structural similarity with the plant PIN-FORMED (PIN) auxin transporter family, while the GPCR-like domain resembles a class B2 GPCR.^12^

Structural studies reveal that LYCHOS operates as a homodimer, with cholesterol binding occurring at a conserved motif located between the GPCR and transporter-like domains.^233,234^ Mutations in the cholesterol-binding motif impair LYCHOS-mediated activation of mTORC1, underscoring its pivotal role in lysosomal cholesterol signaling.^12,232^

Functional analyses show that LYCHOS mediates cholesterol-dependent recruitment of mTORC1 to lysosomes through its interaction with the GATOR1 complex, a key regulator of the Rag GTPase pathway. At high cholesterol levels, LYCHOS sequesters GATOR1, enabling Rag-dependent activation of mTORC1 and promoting anabolic signaling.^232^

Unlike plant PIN transporters, LYCHOS shows reduced affinity for auxin-like molecules such as indole-3-acetic acid (IAA), suggesting its specialization in sensing rather than transport. Consistent with this, SSM-based electrophysiology measurements of LYCHOS revealed that an order-of-magnitude higher concentration of IAA was required to elicit electrogenic currents equivalent to those initiated by PIN8 in plants, consistent with anion binding but likely not with transport across the bilayer.^12^

LYCHOS exemplifies a novel hybrid protein architecture in lysosomes, bridging structural elements from plant transporters and mammalian GPCRs. Its role as a cholesterol sensor underscores the complexity of lysosomal signaling and its importance in maintaining cellular homeostasis.

The table below highlights selected lysosomal uniporters, symporters, and antiporters, emphasizing their substrate specificity, function, and disease associations (Table 3). Please note that this table is by no means exhaustive and shows only those transporters discussed above. Lysosomes harbor a far wider array of SLC transporters, including amino acid transporters [e.g., SLC7A14 (CAT), SLC36A1 (PAT1/LYAAT1), SLC38A7 (SNAT7), SLC38A9], sugar transporters [e.g., SLC2A8 (GLUT8), SLC2A6 (GLUT6), SLC37A2 (SPX2)], nucleoside transporters [e.g., SLC29A3 (ENT3)], metal ion transporters [e.g., SLC11A1 (NRAMP1), SLC40A1 (FPN1), SLC30A2 (ZnT2), SLC46A3 (FKSG16)], lipid transporters (e.g., NPC1), and others. For more details on lysosomal transporters, check out the many excellent reviews on the subject.^4,7,18,160,161^

**Table 3.** Examples of Lysosomal Uniporters, Symporters, and Antiporters

Target	Function	Associated diseases/pathologies	References
SLC15A3 (PHT2)	Proton-coupled di/tripeptide transporter, involved in inflammatory responses and host defense	Inflammation, viral infections, oxidative stress	^162–166^
SLC15A4 (PHT1)	Proton-coupled di/tripeptide transporter; scaffold for TLR7–9 signaling in immune cells	Systemic lupus erythematosus, colitis, psoriasis	^167–172^
SLC17A9 (VNUT)	ATP transporter, purinergic signaling, inflammation	Cancer, disseminated superficial actinic porokeratosis, congenital heart defect (Tetralogy of Fallot), susceptibility to phantom tooth pain	^173–186^
SLC17A5 (Sialin)	Sialic acid transporter, synaptic vesicle function	Sialic acid storage disorders (Salla disease), atherosclerosis, Parkinson’s	^187–198^
SLC64A4 (Cystinosin)	Cystine transporter, nutrient sensing, mTORC1 regulation	Cystinosis, Fanconi syndrome, progressive renal failure	^199–210^
ClC-7 (with OSTM1)	Chloride-proton exchanger, lysosomal acidification	Osteopetrosis, neurodegenerative lysosomal storage disease, myelination defects, albinism, neuropathic pain	^211–219^
TMEM165	Calcium importer, lysosomal calcium homeostasis	Lysosomal function impairment	^221–226^
MFSD1	Dipeptide uniporter, lysosomal amino acid metabolism	Cancer metastasis, immune disorders	^227–231^
LYCHOS (GPR155)	Cholesterol sensor, mTORC1 activation	Disruptions may affect lipid metabolism	^12,232–234^

mTORC1, mechanistic target of rapamycin complex 1.

## Technical Approaches and Advances

Functional studies of lysosomal ion channels, transporters, and pumps have historically been challenging due to lysosomes inaccessibility, their small size, and their complex intracellular environment. Over the years, various technical approaches have been developed to overcome these challenges, allowing researchers to probe the function of these proteins more effectively.

### Electrophysiological characterization of lysosomal channels and transporters at the plasma membrane

As stated earlier, some lysosomal ion channels and transporters are also found in other parts of the cell, including other organelles and the plasma membrane. Lysosomal channels and transporters naturally present at the plasma membrane can be functionally characterized using electrophysiology techniques, such as manual or automated patch clamp.^235,236^ Despite significant differences between lysosomal and plasma membranes, findings from these experiments can, to some extent, be extrapolated to lysosomal-resident channels. Examples include P2X4, BK, VRAC, TMEM63A, TRPM2, TRPA1, CACNA1A, CFTR, and TWIK2 channels, which are found both in lysosomes and at the plasma membrane.

Interestingly, simple overexpression of certain lysosomal channels and transporters has been reported to result in their accumulation at the plasma membrane, allowing for study with conventional or APC techniques.^13,237,238^ Moreover, specific mutations have been shown to alter lysosomal channel trafficking, leading to their expression at the plasma membrane. For example, the varitint-waddler A419P mutation in TRPML3, linked to pigmentation and hearing defects in mice, significantly increases its plasma membrane expression, facilitating the functional study.^239^ Equivalent mutations in TRPML1 and TRPML2 produce large, inwardly rectifying, Ca^2+^-permeable currents at the plasma membrane.^10,240^

Disrupting lysosomal targeting motifs, such as the dileucine signal, can also induce significant plasma membrane expression of ion channels and transporters, allowing functional characterization through patch-clamp and two-electrode voltage clamp (TEVC) techniques. This approach has been used to study various lysosomal ion channels and transporters, such as TPC2, cystinosin, CLC-7, PQLC2, and others.^199,201,213,227,228,241–243^

The obvious advantage of these approaches is that lysosomal channels and transporters, which are otherwise inaccessible to the patch pipette (or patch hole, in the case of planar patch clamp), become accessible. Another significant advantage is that modern APC platforms allow for high-throughput recordings (up to 384 recordings in parallel), thus allowing for rapid screening of drugs targeting lysosomal transport proteins.^13,244^

However, a major limitation is that channels and transporters are studied in a nonlysosomal environment, meaning the results obtained at the plasma membrane may not fully reflect their function inside the cell. Other proteins, such as LAMP-1/2, present on lysosomal membranes, have been shown to influence the function and stability of ion channels and transporters.^48,245^ Additionally, the lipid composition of the lysosomal membrane can affect protein activity, further underscoring the importance of the native membrane environment.^246,247^

For transporters, direct electrophysiological characterization is limited to electrogenic transporters, where transport results in a net movement of electrical charge.^248^ Even for electrogenic transporters, functional characterization via patch-clamp techniques can be challenging, particularly for low-turnover transporters, as the net charge generated by electrogenic transport is significantly lower than that of ion channels. Therefore, while these techniques have been successfully employed to study various intracellular ion channels and transporters, alternative methods may be required for the investigation of low-turnover transporters.^249^

### Lipid bilayer recordings

Another approach to studying the function of lysosomal channels is to reconstitute and record the activity of these channels in a planar lipid bilayer. This technique allows for the examination of channel activity in a controlled environment and is most often used for single-channel studies, providing detailed information about channel gating, conductance, and pharmacology.^250^

Lipid bilayer recordings have been successfully used to study several important lysosomal channels, including TPC2 and TRPML1, revealing critical insights into their conductance, gating properties, and pharmacological sensitivities.^251,252^

While this technique offers powerful insights into the biophysical properties of lysosomal ion channels, it can be technically challenging at every stage—from painting stable bilayers and reconstituting ion channels to data analysis.^253^ Fortunately, modern lipid bilayer recording platforms, such as the Orbit platforms from Nanion Technologies, allow for parallel bilayer recordings (e.g., 4–16 simultaneous recordings), greatly simplifying bilayer formation and increasing the likelihood of successful ion channel incorporation.^254^ These systems are increasingly used to assess organellar ion channels, such as RYR1, and represent an excellent alternative to conventional low-throughput bilayer recording stations.^255^ Apart from the Orbit systems, several academic groups have also reported developing parallel lipid bilayer recording platforms, allowing for up to 96 parallel recordings.^256,257^ And although none of these proof-of-concept systems have been reported as having reached the market, these developments show that there is an interest in having lipid bilayer recordings in high throughput.

It’s worth saying that lipid bilayer recordings are most effective when used in conjunction with other methods, such as conventional electrophysiology and molecular biology approaches, to provide a comprehensive understanding of channel function in the cellular context. This combined approach allows researchers to bridge the gap between isolated channel behavior and physiological relevance.

In addition to planar lipid bilayer recordings, giant unilamellar vesicle (GUV)-based approaches have also become valuable tools for studying organellar channels, including IP3R, RyR, TPC2, PKD2L1, TRPA1, VDAC, and MCU.^258–262^ In practice, membrane fractions enriched with organellar proteins are fused with GUVs or are incorporated into GUVs during the GUV formation process. These GUVs are large enough to be patched using conventional or planar patch clamp setups, providing high-resolution, low-noise recordings of ion channel activity.^258,259^ This method overcomes some of the challenges associated with planar lipid bilayer recordings, as it allows for the study of intracellular channels in a membrane environment that preserves their interaction with partner proteins, while also enabling the use of conventional and APC systems.

### Lysosomal patch clamp

Lysosomal patch clamp is an exciting technique that has transformed how we study ion channels in lysosomes. This approach allows for detailed functional characterization of native enlarged lysosomal channels within their physiological environment, preserving important interactions with endogenous partner proteins.^8–10,263^

Typically, lysosomes are too small to manipulate using traditional patch-clamp techniques, but this challenge has been overcome by genetically or pharmacologically enlarging the organelles. Although one of the most widely used techniques to enlarge endolysosomes is via using vacuolin-1, a few other pharmacological (YM201636, Apilimod, Vicenistatin, Wortmannin + Latrunculin B, sucrose) and genetic (SKD1/VPS4, Rab5-Q79L) tools have also been reported to induce early endosome and endolysosome enlargement.^263–266^ Once enlarged, lysosomes can be released from cells by dissecting plasma membrane and directly patched with manual patch-clamp systems.^8,9^ It should be noted, however, that manual lysosomal patch clamp is technically very demanding and a very low throughput process. Therefore, some efforts have been made to improve the technique in order to make it easier and more amenable to automated planar patch-clamp systems.

A few studies have shown that following enlargement with vacuolin-1, lysosomes can be isolated in bulk from cells using density gradient centrifugation.^8,267–269^ The obtained purified lysosomal preparation is not only amenable to manual patch clamp but also to automated planar patch clamp. Both TRPML1 and TPC2 channels have been studied with the low-throughput semiautomated planar patch clamp using this approach.^267,269,270^ Recent data show that the technique was successfully scaled up to the high-throughput fully APC systems and is now proposed as a service by a few Contract Research Organizations (CROs), including Axxam and SB Drug Discovery.^11^

Given the increased interest from both academia and pharma in studying lysosomes and other intracellular organelles, a few companies are now proposing organelle isolation and purification as a service. One such company is Oria Bioscience (https://www.oriabs.com/), a French startup supplying pharmaceutical laboratories and CROs with purified organelles (lysosomes, mitochondria, and endoplasmic reticulum). Recent promising data show a high success rate in high-throughput APC experiments with lysosomes provided by Oria Bioscience.^271^

**FIG. 1.**
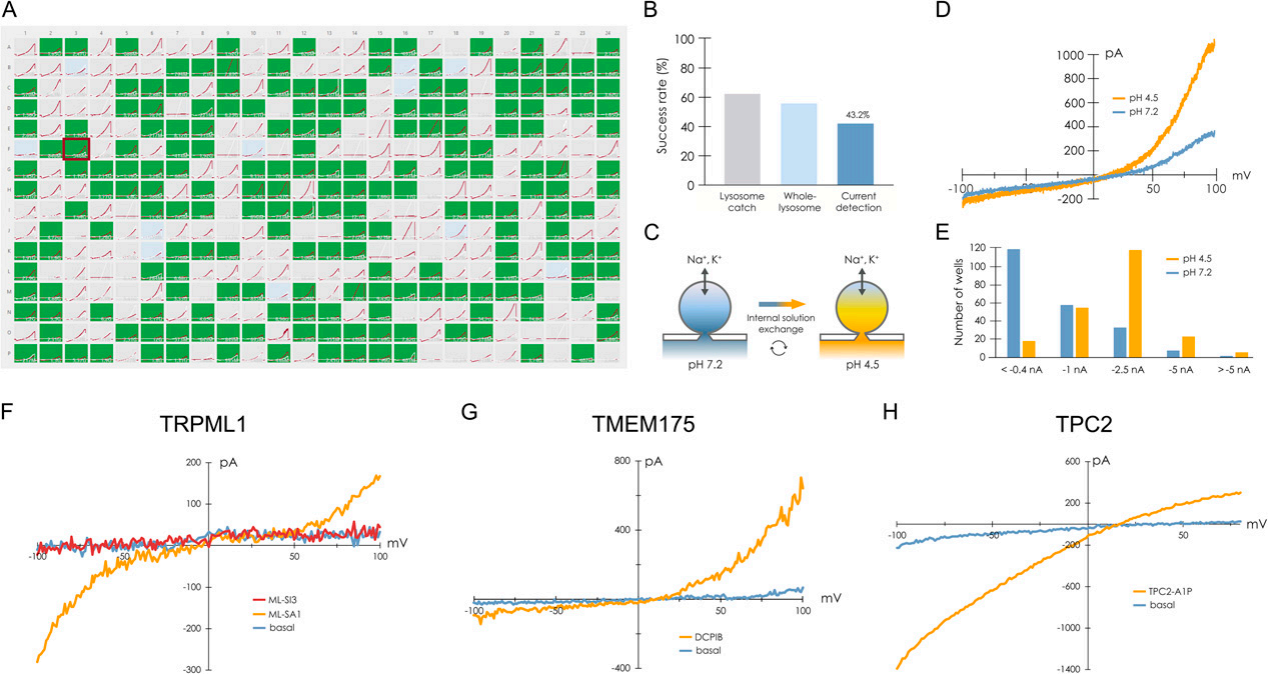
High-throughput automated patch clamp recordings of lysosomal ion channels. **(A)** Example SyncroPatch 384 recording from enlarged (vacuolin-treated) WT lysosomes, shown in a 384-well format. Lysosomes were recorded at a holding potential of 0 mV (+60 mV for TPC2) using a voltage ramp protocol from −100 to 100 mV (to +80 mV for TPC2) over 1 s, applied every 10 s at a sampling frequency of 5 kHz, without leak subtraction. Green color coding indicates wells with Rseal > 200 MΩ. **(B)** Success rate analysis for WT lysosomes, showing a final success rate of 43.2%, with 166 successful recordings in a single run (∼20 min total recording time). Quality filters applied: Rseal > 200 MΩ and current >150 pA at +100 mV. **(C)** Intraluminal solution exchange was performed to assess pH sensitivity during continuous recordings. **(D)** Example SyncroPatch 384 recording from enlarged WT lysosomes, where pH was changed from 7.2 to 4.5; voltage ramp protocol was applied. **(E)** Current distribution at +100 mV, illustrating pH dependence. **(F–H)** Example recordings from lysosomes overexpressing TRPML1, TMEM175, or TPC2, activated by ML-SA1 (or inhibited by ML-SI3), DCPIB, or TPC2_A1P, as indicated. TRPML1, transient receptor potential mucolipin 1; TMEM175, transmembrane protein 175; TPC2, two-pore channel 2.

To evaluate the feasibility of high-throughput automated electrophysiology on isolated lysosomes, we conducted APC experiments using the SyncroPatch 384 platform. Enlarged lysosomes (vacuolin-treated) were provided by Oria Bioscience and tested for their ability to form high-resistance seals (Rseal > 200 MΩ) in whole-lysosome recordings (see Supplementary Data). Our results demonstrated a success rate of 43.2%, with 166 successful recordings in a single run (∼20 min total recording time) (Fig. 1A, B). We performed intraluminal solution exchange during continuous recordings and showed that altering the luminal pH from 7.2 to 4.5 significantly influenced lysosomal ion currents (Fig. 1C–E).

Further, we examined lysosomes overexpressing specific ion channels, including TRPML1, TMEM175, and TPC2, and their response to known activators (ML-SA1, DCPIB, TPC2-A1-P) or inhibitors (ML-SI3 for TRPML1) (Fig. 1F–H). These results highlight the applicability of APC for functional studies of lysosomal ion channels, demonstrating both high data quality and reproducibility in isolated lysosomes.

It is clear that automated patch clamp (APC) offers new opportunities in the study of lysosomal ion channels by enabling high-throughput recordings and eliminating experimenter bias in lysosome selection. Unlike manual patch clamp, which requires optical control to target individual lysosomes, APC relies on suction to capture lysosomes in suspension, streamlining the process and dramatically increasing the speed and scale of data collection. However, the lack of visual control also means that lysosomes cannot be individually repositioned for optimal sealing, representing a trade-off between throughput and fine-tuned control. An important consideration in APC is the use of fluoride in the internal solution during most recordings. While fluoride helps with seal formation, it has also been reported to interfere with ion channel kinetics. This could be particularly relevant for lysosomal recordings involving intraluminal calcium, as fluoride binds calcium. Therefore, this effect should be taken into account when designing and interpreting experiments.

Recent advancements in APC technology have addressed some of these challenges. For instance, developments now allow for APC recordings without fluoride, although further optimizations are needed for certain experimental conditions.^272^ Ongoing research continues to refine APC techniques for lysosomal studies, aiming to combine the benefits of high-throughput capabilities with improved control over recording conditions.

### Solid supported membrane-based electrophysiology (SSME)

**FIG. 2.**
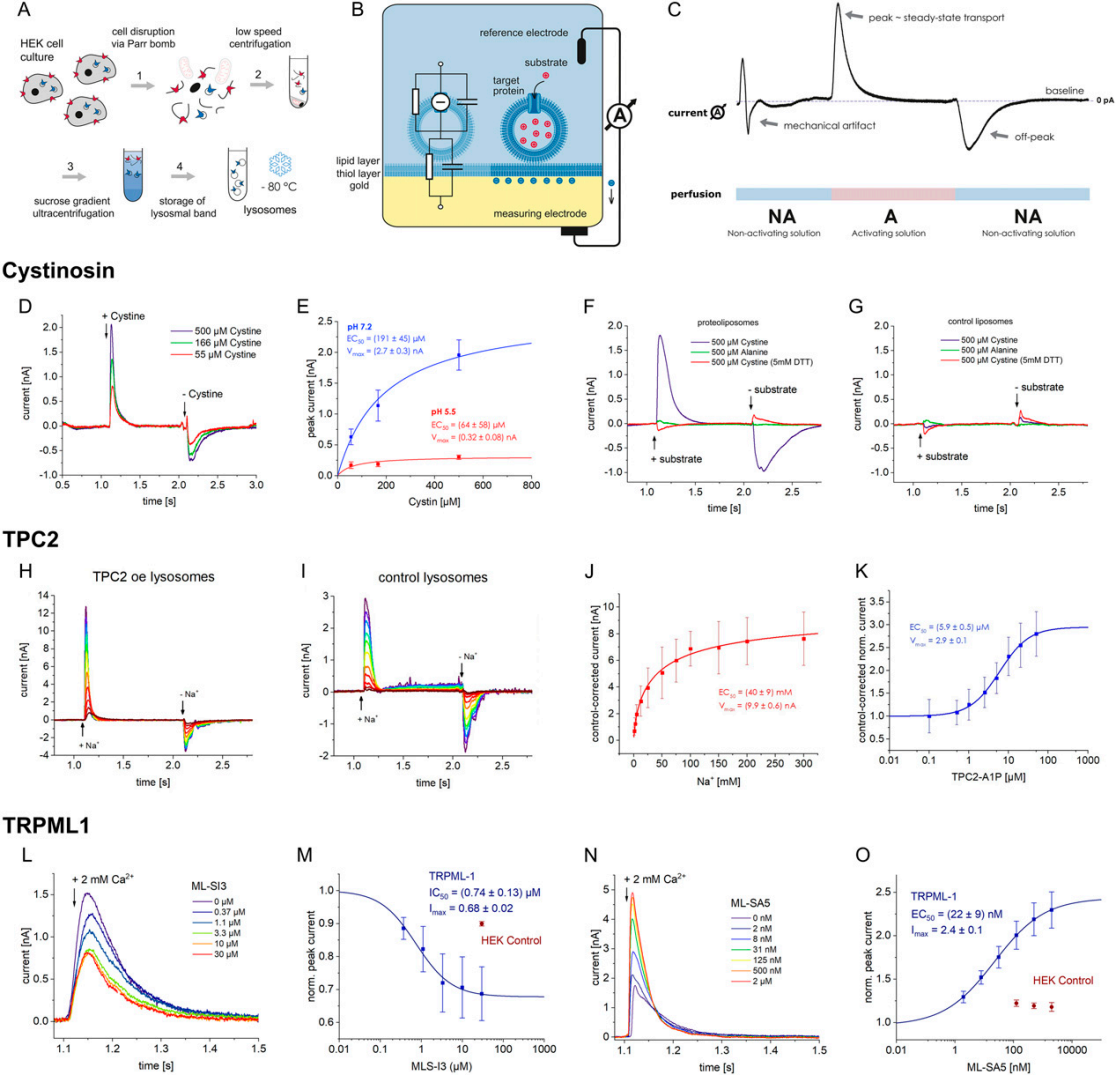
SSME-based electrophysiology for lysosomal transporters: cystinosin, TPC2, and TRPML1. **(A–C)** Principles of SSM-based electrophysiology. **(A)** Schematic representation of the protocol steps for lysosome extraction from HEK293 cell culture. **(B)** Measurement principles of SSM-based electrophysiology. **(C)** Example of a current trace recorded using the SSME platform SURFE²R. **(D–G)** SSME recordings of the lysosomal H^+^/cystine cotransporter cystinosin. Cystinosin was purified and reconstituted into proteoliposomes. Experiments have been carried out using measurement buffers containing 30 mM HEPES, 30 mM MES, 140 mM NaCl, 5 mM MgCl2 titrated to the desired pH using NaOH. **(D)** Current traces demonstrate cystine-stimulated H^+^/cystine influx, with **(E)** dose–response analysis showing reduced EC_50_ and Vmax at acidic pH. **(F)** Control experiments confirm substrate specificity, as alanine and DTT-treated cystine show no significant response. **(G)** Liposomes without cystinosin exhibit no cystine-induced currents, confirming transporter dependency. *n* = 4. **(H–K)** SSME recordings of the lysosomal TPC2. TPC2 was overexpressed in HEK293 cells, and lysosomes have been purified. Experiments have been carried out using measurement buffers containing 30 mM HEPES, 30 mM MES, 300-x mM NMDG-Cl, and x mM NaCl, titrated to pH 7.4 using NMDG. **(H)** Current traces show Na^+^ influx through TPC2 driven by the Na^+^ gradient in the absence of voltage [in TPC2-overexpressing (oe) lysosomes]. **(I)** Negative control lysosomes exhibit near-linear Na^+^ dependence and symmetric peaks, indicating background currents from ion binding to the membrane. **(J)** Na^+^ dose–response analysis revealing current saturation at elevated Na+ concentrations. **(K)** The TPC2-A1P enhancer increases TPC2-mediated flux and dose–response analysis. *n* = 3. **(L–O)** SSME recordings of the lysosomal TRPML1. TRPML1 was overexpressed in HEK293 cells, and lysosomes have been purified. Experiments have been carried out using measurement buffers containing 25 mM HEPES, 25 mM MES, 140 mM NMDG, and 140 mM choline chloride, titrated to pH 7.5 using HCl. Nonactivating solution contained an additional 4 mM choline chloride, while the activating solution was supplemented with 2 mM CaCl_2_. **(L)** ML-SI3 partially inhibits TRPML1, with residual currents at high concentrations reflecting background Ca^2+^ binding to the membrane. **(M)** ML-SI3 showed dose-dependent inhibition, with an IC_50_ of 0.74 µM with minimal effects in control lysosomes. **(N, O)** ML-SA5 displayed dose-dependent potentiation, with an E_50_ of 22 nM. *n* = 3. SSM, solid-supported membrane; SSME, solid-supported membrane-based electrophysiology.

SSME is another powerful tool for studying the activity of electrogenic pumps, transporters, and ion channels, including those residing on lysosomes.^13,249^ SSME is a highly sensitive, cell-free technique that involves reconstituting purified proteins or membrane vesicles containing pumps, transporters, or ion channels onto an SSM sensor. This sensor detects transient currents resulting from electrogenic transport processes, allowing for real-time measurements of pump/transporter/ion channel activity^249,273,274^ (Fig. 2A–C).

SSME has proven particularly valuable for investigating low-turnover transporters, which, as we mentioned earlier, are often challenging to study with traditional electrophysiology techniques. The large surface area of SSME sensors, combined with the ability to measure signals from millions of vesicles simultaneously, significantly enhances the signal-to-noise ratio, making it possible to detect even subtle transport events. It should be noted, however, that unlike patch clamp, SSME does not allow control over the membrane voltage, with currents driven by chemical gradients rather than the applied voltage.^249,274^

Another advantage of SSME is its versatility. The technique can be applied to membrane preparations from a wide range of sources, including cell lines, tissues, and even plants or bacteria. This flexibility allows researchers to study proteins in their physiological context, and therefore SSME is an excellent technique for studying intracellular ion channels and transporters in their natural environment, preserving crucial interactions with partner proteins and maintaining physiological conditions.^275,276^

While SSME is a highly versatile and sensitive approach, certain experimental considerations should be noted. Since SSME relies on detecting transient currents driven by chemical gradients, it is particularly well suited for transporters, pumps, and ligand-gated ion channels but is not applicable to voltage-gated channels, which require direct voltage control. Additionally, while SSME enhances signal detection by integrating measurements from millions of vesicles, extremely slow transporters—such as certain ABC transporters with turnover rates of only a few molecules per minute—may still fall below the detection limit. Another factor to consider is that some substrates, particularly metal ions and multiaromatic compounds, can generate background currents due to nonspecific membrane interactions, which may require careful assay optimization. Despite these considerations, ongoing technical advancements continue to expand the range of targets that can be effectively studied using SSME, further solidifying its role as a valuable tool in lysosomal ion channel and transporter research.

At present, over 100 different pumps, transporters, and ion channels, including several lysosomal ones, have been studied using SSME. The lysosomal targets include V-ATPase, SLC15A4 (PHT1), ClC-7, and TMEM175.^13,277–279^ Other endosomal targets that have been validated on SSME include the electroneutral Na^+^/H^+^ exchanger NHE9.^280^

To expand the list of lysosomal targets validated for SSME, we conducted a series of experiments using the SURFE²R N1 (Surface Electrogenic Event Reader) platform from Nanion Technologies. We measured the activity of three additional lysosomal proteins—TRPML1, TPC2, and SLC64A4 (cystinosin) (Fig. 2 and Supplementary Data).

Cystinosin-mediated H^+^/cystine cotransport was confirmed in proteoliposomes, showing positive transient currents indicative of charge influx (Fig. 2D). Transport was pH-dependent, with reduced EC^50^ and I_max_ at acidic pH (symmetrical pH conditions), likely due to internal acidification locking the transporter in an inside-open conformation and slowing H^+^ release (Fig. 2E). Control experiments with alanine and dithiothreitol (DTT)-treated cystine showed no significant currents, confirming substrate specificity (Fig. 2F). Additionally, liposomes without cystinosin showed no cystine-induced currents, ruling out nonspecific effects (Fig. 2G). These findings demonstrate that cystinosin specifically transports cystine in a pH-dependent manner, with reduced efficiency under acidic conditions.

TPC2 was overexpressed in human embryonic kidney (HEK) cells, and purified lysosomes showed Na^+^ dose-dependent currents, indicating Na^+^ influx (Fig. 2H). Control lysosomes exhibited smaller, more linear currents with symmetric on- and off-currents, suggesting background ion binding rather than ion flux through the membrane (Fig. 2I). Background currents were consistent with previously published TMEM175 assays, confirming their nonspecific nature.^13^ By subtracting the control currents, we isolated the TPC2-specific response. When plotted against Na^+^ concentration, this response showed saturation kinetics with an EC^50^ of 40 mM, unlike TMEM175, which exhibited a linear response (Fig. 2J). Assay validation included testing the TPC2 enhancer TPC2-A1-P, which, at 5 mM Na^+^, showed an EC^50^ of 6 µM and a maximum potentiation of 290% of the initial flux rate, confirming its effectiveness (Fig. 2K).

Lastly, lysosomes overexpressing TRPML1 were studied to assess the pharmacology of the inhibitor ML-SI3 and enhancer ML-SA5. Using Ca^2+^ concentration jumps, ML-SI3 showed dose-dependent inhibition, with an IC^50^ of 0.74 µM, close to literature values^281,282^ (Fig. 2L, M). However, even at high doses, a residual current remained, attributed to background Ca^2+^ binding to the membrane, as also recorded with control lysosomes (data not shown). ML-SA5 displayed dose-dependent potentiation, with an EC^50^ of 22 nM, suggesting a higher apparent affinity than previously reported (333 nM in vacuoline-treated lysosomes)^283^ (Fig. 2N, O). The maximum potentiation of TRPML1 net current was ∼7.5, confirming its strong activation. Both ML-SI3 and ML-SA5 had minimal effects on control lysosomes, demonstrating assay specificity.

Recent advancements have made SSME higher throughput. The new high-throughput SSME platform from Nanion Technologies, called SURFE²R 96SE, has dramatically increased the data throughput, enabling researchers to conduct high-throughput screening assays. This capability has sparked particular interest from pharmaceutical companies and CROs looking to identify and characterize new drug candidates targeting ion channels and transporters. Several CROs, including WuXi, SB Drug Discovery, Evotec, and others, already offer SSME studies as a service, utilizing both low (SURFE^2^R N1) and high-throughput (SURFE²R 96SE) platforms. An increasing number of transporters are being assayed using SSME, which now ranks 3rd among 33 different SLC functional assays in terms of the number of validated assays by the RESOLUTE consortium.^284,285^

### Lysosomal imaging

Lysosomal imaging has emerged as a powerful tool for functional studies of lysosomal ion channels and transporters, allowing researchers to monitor real-time changes in ion concentrations, pH, and membrane potential in a subcellular context.^14,15^ Early on, radiolabeled flux or uptake assays were used for measuring ion fluxes through ion channels and transporters in membrane vesicles.^211,248^ Then fluorescence assays have become popular, allowing to monitor the activity of ion channels and transporters in cells in real time.^286,287^ More recently, genetically encoded indicators have revolutionized this field by enabling the targeting of fluorescent or luminescent probes specifically to lysosomes, providing insights into the activity of lysosomal ion channels and transporters.^14,15,288^

One widely used approach involves organelle-targeted genetically encoded Ca^2+^ indicators (GECIs), which are designed to monitor calcium dynamics. GECIs, such as GCaMPs, consist of a Ca^2+^-binding motif fused to a fluorescent reporter, allowing for a direct readout of calcium concentration through changes in fluorescence.^288–291^ These indicators can be precisely targeted to the lysosome by fusing them with lysosomal-specific targeting sequences, ensuring that the functional dynamics of lysosomal Ca^2+^ channels and transporters, like TRPML1 or TMEM165 can be studied in their native environment.^15,221,292,293^

Beyond calcium, lysosomal imaging has expanded to include H^+^, K^+^, Cl^−^, and Na^+^-sensitive indicators, enabling the exploration of a variety of ion transport activities in lysosomes.^294–297^ Moreover, organelle-targeted genetically encoded voltage indicators and DNA-based voltage indicators have also been developed to measure changes in the membrane potential of lysosomes, offering a deeper understanding of how ion channels and pumps like V-ATPase contribute to lysosomal voltage regulation.^298^

These imaging techniques, combined with genetic manipulations such as knockout or overexpression models, allow researchers to explore the physiological roles of lysosomal ion channels and transporters in a highly specific manner.

## Conclusions and Future Perspectives

The study of lysosomal ion channels, transporters, and pumps has evolved significantly, progressing from early radiolabeled flux assays to modern, advanced techniques such as lysosomal patch clamp, lysosomal imaging, APC and SSME. These technological advancements have greatly expanded our understanding of lysosomal function and disease mechanisms, positioning lysosomal transport proteins as important therapeutic targets. Once considered inaccessible, lysosomal ion channels and transporters are now being studied with medium- to high-throughput APC and SSME, enabling systematic drug discovery efforts at an unprecedented scale.

As technology continues to evolve, the field of lysosomal transport is set to provide deeper insights into the roles these proteins play in both normal physiology and pathological conditions. The recent discovery of new lysosomal transport proteins, such as TMEM165, along with newly identified roles for established proteins like ATP13A2, exemplifies the ongoing expansion of our knowledge in this area.

However, despite significant progress, several fundamental questions remain unresolved. The functional properties of many lysosomal channels and transporters require further investigation. The exact structural determinants governing their ion permeability, as well as their dynamic regulation by cellular signals, remain major open questions. Advances in cryo-EM, patch-clamp electrophysiology, and computational modeling are expected to provide deeper insights into these mechanisms, ultimately aiding in the development of targeted therapeutics.

Our bibliometric analysis shows that organellar ion channels are currently the second fastest-growing field in ion channel research, surpassed only by mechanosensitive channels. Increased interest from the pharmaceutical industry is further driving advancements in this field. A recent notable acquisition by Merck of Caraway Therapeutics, which had TRPML1 and TMEM175 modulators in its pipeline, highlights this trend. Additionally, Autifony Therapeutics and Boehringer Ingelheim are collaborating on an undisclosed lysosomal ion channel target, aiming to develop first-in-class modulators with the potential to treat a range of indications linked to lysosomal dysfunction. Also, SiSaf Ltd. is pioneering a novel siRNA therapeutic to target and suppress mutant CLCN7 in autosomal dominant osteopetrosis type 2 (ADO2) patients.

It is also exciting to witness the emergence of new companies dedicated to developing modulators targeting lysosomal ion channels, including Lysoway Therapeutics and Threebrooks Therapeutics, both founded after 2020, and the recently launched Tenvie Therapeutics, which has secured $200M to advance drug development for lysosomal TRPML1 and TMEM175.

The development of small-molecule activators and inhibitors over the past two decades has been instrumental in defining the roles of lysosomal ion channels and transporters in lysosomal signaling, autophagy, and disease. These tool compounds not only facilitated basic research but also opened new possibilities for drug discovery, transforming previously inaccessible lysosomal targets into viable therapeutic candidates. But, despite these promising advancements, the potential off-target effects of pharmacological modulators remain an important question requiring special attention. Furthermore, membrane-permeable drugs often alter the physicochemical properties of biological membranes, especially at higher doses above 10 µM. SSME can track these nonspecific effects by measuring capacitive currents resulting from ion-membrane interactions. This capability allows for evaluating dose thresholds while also assessing the drug’s specificity and efficacy for the target protein.

Future research should aim to improve the specificity and safety of lysosomal ion channel modulators, utilizing structure-based drug design, AI-assisted screening, and *in vitro* validation in physiologically relevant models such as patient-derived organoids and induced pluripotent stem cell (iPSC)-based systems. A better understanding of cell-type-specific effects of lysosomal ion transport modulation will also be crucial, as lysosomal channels exhibit differential expression patterns across tissues and disease states. Furthermore, given the growing recognition of lysosomal dysfunction in neurodegenerative diseases such as Parkinson’s and Alzheimer’s, it is imperative to explore in more detail how lysosomal ion homeostasis interacts with pathways involved in protein aggregation, autophagy, and neuronal survival.

Collaboration between established biotech companies, academic labs, CROs, and startups specializing in organellar research is driving innovation and the development of cutting-edge tools that accelerate both fundamental research and drug discovery efforts. In this regard, it is particularly worth mentioning the collaborative efforts of Nanion Technologies, Ludwig-Maximilians-University Munich, University of Regensburg, SB Drug Discovery, Cerevel Therapeutics, Assay Works, AXXAM, and Oria Bioscience to develop high-throughput screening assays for lysosomal channels and transporters.^11,13^

The ongoing integration of academic research, industry-driven drug development, and startup-driven innovation will be crucial for translating basic discoveries into effective therapeutic strategies for diseases driven by lysosomal dysfunction. As research in this field progresses, we anticipate the emergence of highly selective, clinically viable treatments for a range of lysosomal disorders, neurodegenerative diseases, and cancers linked to lysosomal dysfunction. However, achieving this goal will require sustained investment in selectivity-driven drug design, disease modeling, and multi-omics approaches to fully elucidate the systemic impact of lysosomal ion transport modulation.
