# Supplementary material for: Lysosomal Ion Channels and Transporters: Recent Findings, Therapeutic Potential, and Technical Approaches
Source: Bioelectricity. 2025 Mar 18;7(1):29–57. doi: 10.1089/bioe.2025.0010 (PMC12056583; doi:10.1089/bioe.2025.0010)
Supplement: Supplementary Data [file bioe.2025.0010_supplementary_data.docx]

**Supplementary Material**

**Isolation of lysosomes for automated patch clamp (APC) recordings**

Enlarged lysosomes (1 µM Vacuolin) were provided by Oria Bioscience and isolated from HEK cells as described by Santhino et al.^1^ The samples were shipped on dry ice and used immediately on the SyncroPatch 384 at a density of 200,000 lysosomes per mL.

**Electrophysiology – automated patch clamp (APC)**

Whole-cell patch-clamp recordings were conducted according to Nanion’s standard procedures for the SyncroPatch 384. All recordings were performed at room temperature (21°C) using planar borosilicate glass chips, with NPC-384T nanoS-type (1×; PN: 22 2111) consumables being critical for experimental success. These consumables were specifically designed to support and maintain lysosomal integrity during recordings.

To optimize performance, we implemented a low-density lysosome approach, reducing both lysosome count and volume while maintaining a high catch rate. This method, combined with the specialized consumable, enabled stable lysosomal seals in ‘whole-lysosome’ mode, with seal resistances consistently exceeding 0.2 GΩ. This stability was crucial for enabling intraluminal solution exchange (Figure 1).

Lysosomes were recorded at a holding potential of 0 mV (+60 mV for TPC2) using a voltage ramp protocol from −100 to 100 mV (to +80 mV for TPC2) over 1 s, applied every 10 s at a sampling frequency of 5 kHz, without leak subtraction.

For TPC2 the bath/cytoplasmic solutions contained (mM): K-Gluconate (130), KCl (10), NaCl (4); MgCl_2_ (2), CaCl_2_ (2), HEPES (20), pH 7.2 whereas luminal solutions contained (mM): NaF (110); NaCl (20), EGTA (10), HEPES (10), pH 7.2.

For TMEM175/TRPML1 the bath/cytoplasmic solutions contained (mM): KCl (120), NaCl (10); Glucose (5) MgCl_2_ (2), CaCl_2_ (2), HEPES (10), pH 7.2 whereas luminal solutions contained (mM): CsF (110); CsCl (10), NaCl (20), EGTA (10), HEPES (10), pH 7.2.

Data was not corrected for liquid junction potential (ljp ~14 mV for K-Gluconate in our settings) that led to underestimated reversal potentials (E_rev_) for the TPC2 recording. For experiments with intraluminal pH 4.5 solutions, HEPES was exchanged by MES. Data acquisition was performed using PatchControl 384 (Nanion Technologies) software.

**Isolation of lysosomes for SSME recordings**

Lysosomes were isolated following a protocol adapted from Schulz et al.^2^ HEK293 cells were lysed, and the lysate was centrifuged at 6000× g for 10 min at 4°C to remove cell debris. The supernatant was centrifuged at 100,000× g for 30 min at 4°C to collect membrane fractions. The resulting pellet was resuspended in disruption buffer (up to 3 mL) for further purification.

Lysosomal enrichment was performed using sucrose gradient ultracentrifugation. Sucrose solutions of 9%, 31%, 45%, and 70% (w/v) in 10 mM Tris, pH 7.5 were prepared. The membrane pellet was resuspended in 70% sucrose and ultracentrifuged at 100,000× g for 18 h at 4°C.

Following centrifugation, lysosomal fraction (31%/45% interface) was collected, diluted 4x in storing buffer (30 mM HEPES, pH 7.5, 140 mM NMDG-Cl, 2 mM MgCl₂, 0.2 mM DTT, 5% glycerol) and centrifuged at 100,000× g for 30 min at 4°C. The pellet was resuspended in ~200 µL storing buffer, aliquoted into 10 µL portions, flash-frozen in liquid nitrogen, and stored at −80°C until use.

All subsequent SSME recordings were performed on native, non-treated lysosomes.

**Solid supported membrane-based electrophysiology (SSME)**

SSME recordings were performed under continuous solution flow with the SURFE2R N1 instrument as described previously.^3,4^ In brief, SURFE2R N1 sensors with 3 mm diameter were prepared using a standard pipetting procedure to form the Solid Supported Membrane (SSM) on the gold coated sensor chip. During the process each sensor is filled with 100 µl of non-activating solution, which differed for each of the assays. Detailed buffer compositions are provided below.

For Cystinosin recordings, the transporter was purified and reconstituted into proteoliposomes with a POPE:POPG ratio of 3:1 and a lipid-to-protein ratio (w/w) of 10. Experiments have been carried out using measurement buffers containing 30 mM HEPES, 30 mM MES, 140 mM NaCl, 5 mM MgCl2, titrated to the desired pH using NaOH. Each sensor was prepared by adding ~1 µg of sample, reflecting 0.1 µg of Cystinosin.

TPC2 was overexpressed in HEK293 cells; lysosomes have been purified as described before.^5^ Experiments have been carried out using measurement buffers containing 30 mM HEPES, 30 mM MES, 300-x mM NMDG-Cl and x mM NaCl, titrated to pH 7.4 using NMDG. Each sensor was prepared by adding ~0.5 µg total protein as determined using a Bradford assay. The non-activating solution flushed before 1.1 s and after 2.1 s in time contained 0 mM NaCl, while the activating solution contained 1 < x < 300 mM NaCl.

TRPML1 was overexpressed in HEK293 cells; lysosomes have been purified as described before.^5^ Experiments have been carried out using measurement buffers containing 25 mM HEPES, 25 mM MES, 140 mM NMDG, 140 mM Choline Chloride, titrated to pH 7.5 using HCl. Non-activating solution contained an additional 4 mM choline chloride, while activating solution was supplemented with 2 mM CaCl_2_. Each sensor was prepared by adding ~0.5 µg total protein as determined using a Bradford assay.

10 µl of pre-diluted and sonicated samples, either proteoliposomes or purified lysosomes, were added to each sensor. The sample membrane adsorbs to the SSM and forms a capacitively coupled membrane system. After centrifugation (30 min, 2,500 g) the sensors were ready to use. All measurements were carried out using a standard single solution exchange workflow. The flow sequence contained 1 s non-activating solution (B1), 1 s activating solution (A) and 1 s non-activating solution (B2). In all experiments, the A solutions matched the B solutions except for the presence of the substrate specific to the target protein. Solution A typically contained 500 µM Cystin, 2 mM Ca2+, or 5 mM Na+ for the Cystinosin, TRPML1 and TPC2 assay, respectively. During A flow the on-peak is detected, reflecting the flux rate of each target protein, stimulated by the concentration gradient alone, in the absence of voltage. Enhancers and blockers were applied by rinsing the sensor with 1 mL of non-activating solution and incubating for 3 minutes before measurement. The compounds were also present in both the non-activating and activating solutions during the measurement.

**References**

1. Santinho A, Carpentier M, Lopes Sampaio J, et al. Giant organelle vesicles to uncover intracellular membrane mechanics and plasticity. Nat Commun 2024;15(1); doi: 10.1038/S41467-024-48086-7.

2. Schulz P, Werner J, Stauber T, et al. The G215R mutation in the Cl-/H+-antiporter ClC-7 found in ADO II osteopetrosis does not abolish function but causes a severe trafficking defect. PLoS One 2010;5(9):1–9; doi: 10.1371/JOURNAL.PONE.0012585.

3. Bazzone A, Barthmes M, Fendler K. SSM-Based Electrophysiology for Transporter Research. Methods Enzymol 2017;594:31–83; doi: 10.1016/BS.MIE.2017.05.008.

4. Bazzone A, Barthmes M. Functional Characterization of SLC Transporters Using Solid Supported Membranes. Methods Mol Biol 2020;2168:73–103; doi: 10.1007/978-1-0716-0724-4_4.

5. Bazzone A, Barthmes M, George C, et al. A Comparative Study on the Lysosomal Cation Channel TMEM175 Using Automated Whole-Cell Patch-Clamp, Lysosomal Patch-Clamp, and Solid Supported Membrane-Based Electrophysiology: Functional Characterization and High-Throughput Screening Assay Development. Int J Mol Sci 2023;24(16); doi: 10.3390/IJMS241612788.
